# The Emirates Mars Mission (EMM) Emirates Mars InfraRed Spectrometer (EMIRS) Instrument

**DOI:** 10.1007/s11214-021-00848-1

**Published:** 2021-09-22

**Authors:** Christopher S. Edwards, Philip R. Christensen, Greg L. Mehall, Saadat Anwar, Eman Al Tunaiji, Khalid Badri, Heather Bowles, Stillman Chase, Zoltan Farkas, Tara Fisher, John Janiczek, Ian Kubik, Kelly Harris-Laurila, Andrew Holmes, Igor Lazbin, Edgar Madril, Mark McAdam, Mark Miner, William O’Donnell, Carlos Ortiz, Daniel Pelham, Mehul Patel, Kathryn Powell, Ken Shamordola, Tom Tourville, Michael D. Smith, Nathan Smith, Rob Woodward, Aaron Weintraub, Heather Reed, Emily B. Pilinski

**Affiliations:** 1 Department of Physics and Astronomy, Northern Arizona University, NAU BOX 6010, Flagstaff, AZ 86011 USA; 2 School of Earth and Space Exploration, Arizona State University, Tempe, AZ USA; 3 Mohammed bin Rashid Space Center, Emirates Institute for Advanced Science and Technology, Al Khawaneej Area, Dubai, UAE; 4 Arizona Space Technologies, Tempe, AZ USA; 5 Goddard Space Flight Center, Greenbelt, MD USA; 6 Laboratory for Atmospheric and Space Physics, University of Colorado Boulder, Boulder, CO USA

**Keywords:** Mars, Atmosphere, EMM, Emirates Mars Infrared Spectrometer

## Abstract

The Emirates Mars Mission Emirates Mars Infrared Spectrometer (EMIRS) will provide remote measurements of the martian surface and lower atmosphere in order to better characterize the geographic and diurnal variability of key constituents (water ice, water vapor, and dust) along with temperature profiles on sub-seasonal timescales. EMIRS is a FTIR spectrometer covering the range from 6.0-100+ μm (1666-100 cm^−1^) with a spectral sampling as high as 5 cm^−1^ and a 5.4-mrad IFOV and a 32.5×32.5 mrad FOV. The EMIRS optical path includes a flat 45° pointing mirror to enable one degree of freedom and has a +/- 60° clear aperture around the nadir position which is fed to a 17.78-cm diameter Cassegrain telescope. The collected light is then fed to a flat-plate based Michelson moving mirror mounted on a dual linear voice-coil motor assembly. An array of deuterated L-alanine doped triglycine sulfate (DLaTGS) pyroelectric detectors are used to sample the interferogram every 2 or 4 seconds (depending on the spectral sampling selected). A single 0.846 μm laser diode is used in a metrology interferometer to provide interferometer positional control, sampled at 40 kHz (controlled at 5 kHz) and infrared signal sampled at 625 Hz. The EMIRS beamsplitter is a 60-mm diameter, 1-mm thick 1-arcsecond wedged chemical vapor deposited diamond with an antireflection microstructure to minimize first surface reflection. EMIRS relies on an instrumented internal v-groove blackbody target for a full-aperture radiometric calibration. The radiometric precision of a single spectrum (in 5 cm^−1^ mode) is <3.0×10^−8^ W cm^−2^ sr^−1^/cm^−1^ between 300 and 1350 cm^−1^ over instrument operational temperatures (<∼0.5 K NE$\Delta $T @ 250 K). The absolute integrated radiance error is < 2% for scene temperatures ranging from 200-340 K. The overall EMIRS envelope size is 52.9×37.5×34.6 cm and the mass is 14.72 kg including the interface adapter plate. The average operational power consumption is 22.2 W, and the standby power consumption is 18.6 W with a 5.7 W thermostatically limited, always-on operational heater. EMIRS was developed by Arizona State University and Northern Arizona University in collaboration with the Mohammed bin Rashid Space Centre with Arizona Space Technologies developing the electronics. EMIRS was integrated, tested and radiometrically calibrated at Arizona State University, Tempe, AZ.

## Introduction

The Emirates Mars Infrared Spectrometer (EMIRS) instrument onboard the Emirates Mars Mission (EMM) dubbed “Hope” was launched to Mars on 19 July 2020 at 21:58:14 UTC (20 July 2020 06:58:14 JST) from the Tanegashima launch site in Japan. EMIRS will aid the mission goals of EMM by characterizing the state of the lower atmosphere of Mars through systematic observations that enable near-complete geographic coverage over the full martian day on sub-seasonal timescales from its 20,000–43,000 km elliptical orbit More specifically, the science objectives of the EMIRS investigation are to: 1) Determine the three-dimensional thermal state of the lower atmosphere and its diurnal variability on sub-seasonal timescales, 2) Determine the geographic and diurnal distribution of key constituents in the lower atmosphere on sub-seasonal timescales.

**Fig. 1:**
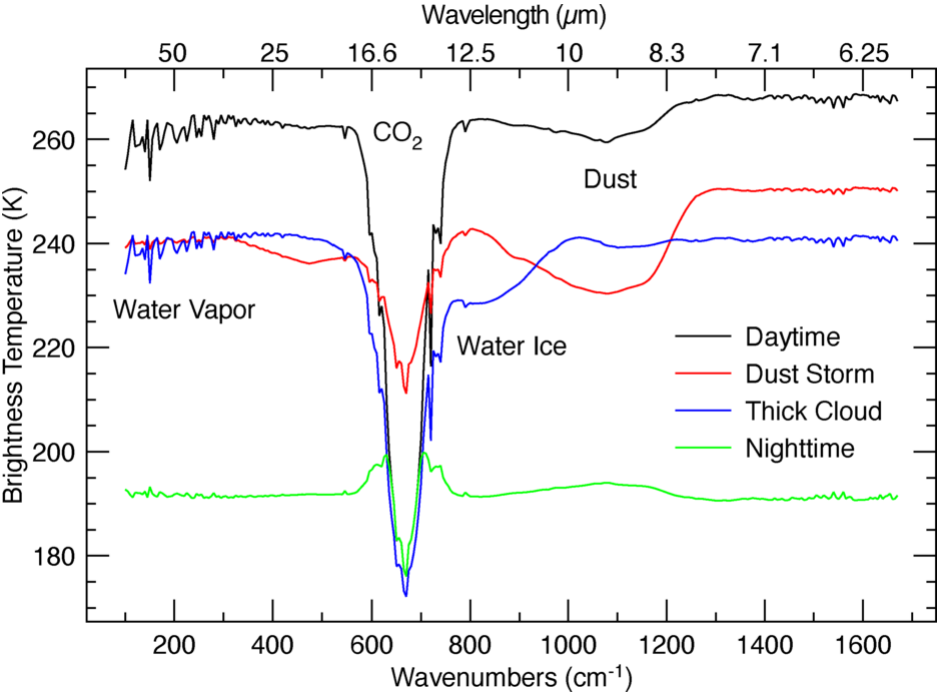
Synthetic spectra of the martian atmosphere simulating what will be observed by EMIRS with 5 cm^−1^ sampling over the EMIRS spectral range. Various conditions are illustrated demonstrating the expected variability in EMIRS observations. Daytime corresponds to local noon and nighttime corresponds to local midnight

These instrument investigations are carried out using thermal infrared observations of the martian disk from 1666 to 100 cm^−1^ (6 to 100 μm). The driving requirements for the EMIRS instrument are the spectral range, sampling, radiometric precision, absolute accuracy and instantaneous field of view, all of which enable the unique CONOPS of the EMM mission at Mars. Figure 1 provides an example set of model spectra (in brightness temperature) that illustrate the key atmospheric parameters EMIRS will derive. These data, all acquired nadir looking (e.g. no limb observations), will enable the determination of the column integrated abundance of atmospheric water vapor (Smith 2002), the column integrated dust and water ice opacities (Smith 2004), and the atmospheric temperature profile as derived using the CO_2_ absorption feature at ∼15 μm (Conrath et al. 2000) (Fig. 1).

Atmospherically corrected surface temperatures will also be derived and will enable supplementary thermophysical investigations building on methods used by other authors (e.g. thermal inertia (Edwards et al. 2009; Mellon et al. 2000; Putzig and Mellon 2007a, 2007b; Putzig et al. 2005), block abundance (Christensen 1986; Kieffer et al. 1976; Nowicki and Christensen 2007), CO_2_ frost formation (Piqueux et al. 2016). These types of investigations, while not detailed in this paper, will benefit from the use of the complete diurnal coverage provided by EMIRS which can be compared to thermal models (e.g., Kieffer 2013) that predict the surface temperature response as a function of the diurnal input and surface properties, such as block abundance, lateral mixing, vertical layering, etc. Surface emissivity spectra will also be derived by fitting Planck functions to the observed spectra and dividing by the calibrated radiance spectra following the same methods used by the widely successful TES mission (e.g., Bandfield et al. 2000; Christensen et al. 2001; Rogers et al. 2007; Rogers and Christensen 2007). The EMIRS instrument will acquire 5 cm^−1^ spectral sapling data of the martian surface and atmosphere to 100 μm, unlike that captured by previous infrared instruments (e.g. TES, Mars Climate Sounder, Viking’s Infrared Thermal Mapper, etc.). In this paper, we present the detailed description of the as-built EMIRS instrument, an overview of the calibration methodology and performance, as well as an overview of uncertainties related to the retrievals of martian atmospheric properties. Further, we provide an overview of the concept of operations, data processing strategy and plans for public release and archival of the data generated by EMIRS.

## Instrument Description

### Instrument Overview

**Fig. 2:**
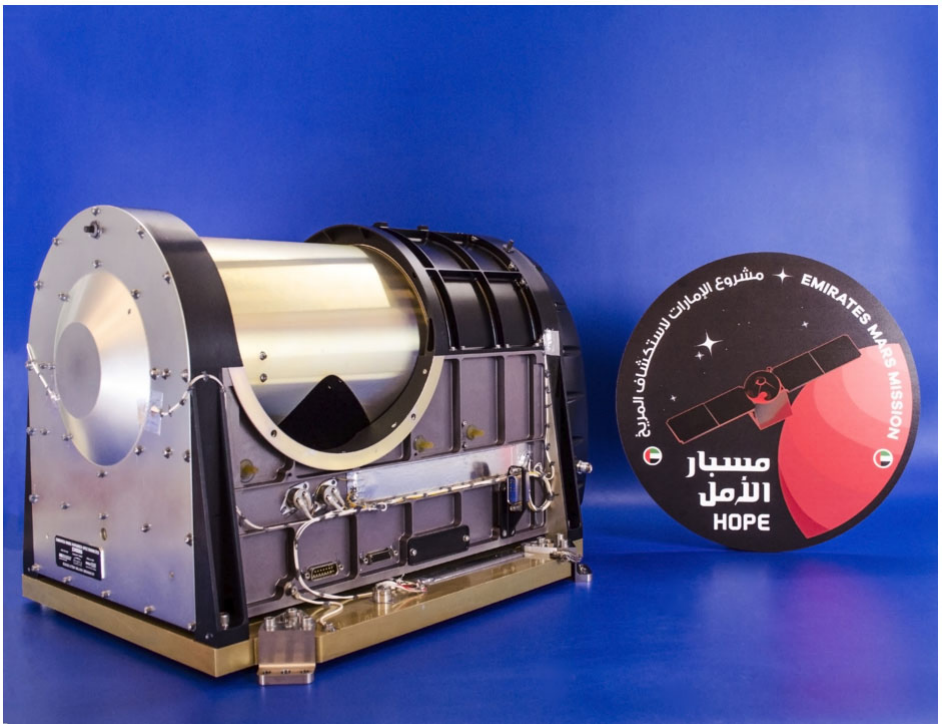
EMIRS Flight instrument prior to integration with the spacecraft

The EMIRS instrument (Fig. 2) continues the heritage line of infrared spectrometers developed by ASU for use on interplanetary missions, including Mars Observer (Christensen et al. 1992), Mars Global Surveyor (MGS) (Christensen et al. 2001), Mars Exploration Rovers (MER) Miniature Thermal Emission Spectrometers (Christensen et al. 2003) and the OSIRS-REx Thermal Emission Spectrometer (OTES) (Christensen et al. 2018). EMIRS is the latest instrument in this long heritage of FTIR spectrometers and returns to flight some of the capabilities that were originally developed for TES (e.g. pointing mirror) more than 25 years ago and boasts the largest telescope diameter of any ASU instrument built to-date. Table 1 provides a complete comparison of all the FTIR instruments designed and flown by ASU on interplanetary missions. EMIRS uses an uncooled DLaTGS detector, which significantly lowers complexity as compared to mechanically cooled detectors, increases longevity, and lowers power consumption. There are numerous advantages of this style of detector, though they all come at the expense of instrument radiometric performance.

**Table 1:** Comparison of the key technical resources and functionality of ASU built interferometer-based instruments

Instrument	Year launched	Spectral range	Spectral sampling	# Bands	IFOV (mrad)	Telescope diameter (cm)	Mass (kg)	Operational power (W)	Size (cm)	Pointing capability
MO/MGS TES	1992/1996	148-1651 cm-1	9.8, 4.9 cm-1	143; 286	8.3	15.20	14.40	14.5	23.6 × 35.5 × 40	Yes, Internal
MER Mini-TES	2003	340-1998 cm-1	9.99 cm-1	167	20	6.35	2.40	5.6	23.5 × 16.3 × 15.5	Yes, via rover mast
OTES	2016	100-1750	8.66 cm-1	193	6.5	15.20	6.27	10.8	37.5 × 28.9 × 52.2	No
EMIRS	2020	100-1666	10 cm-1, 5 cm-1	170; 340	5.4	17.78	14.72	22.4	52.9 × 37.5 × 34.6	Yes, Internal

**Fig. 3:**
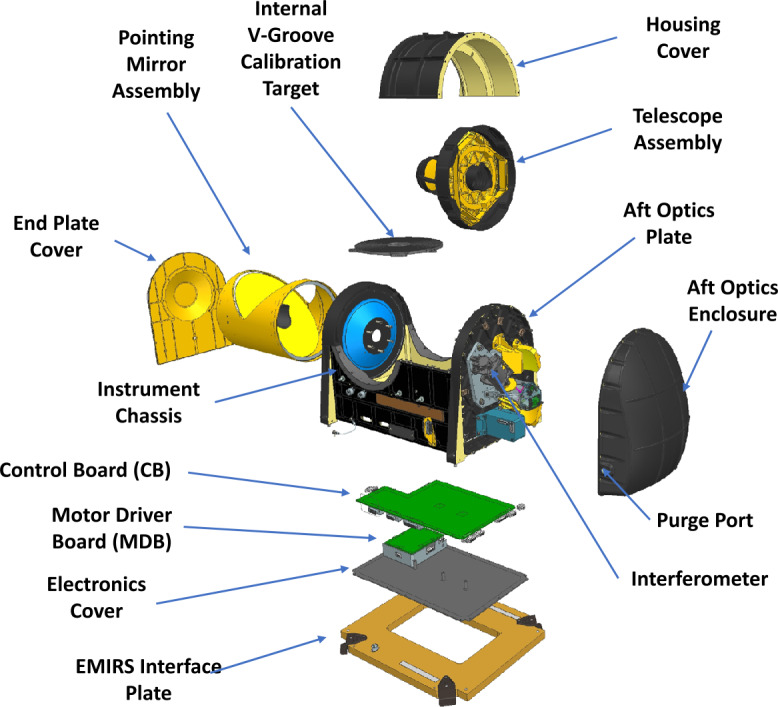
EMIRS Instrument CAD model exploded view with main sub-assemblies labeled

EMIRS collects hyperspectral thermal infrared data over the spectral range from 1666 to 100 cm^−1^ (6 to 100 μm) with a selectable spectral sampling of 5 or 10 cm^−1^ depending on the measurement goal. EMIRS (Fig. 3) uses an array of the aforementioned DLaTGS detectors all with ∼5.5-5.7 mrad FWHM IFOV to achieve a balance between coverage and performance requirements, with the center detector being the highest performing detector. The EMIRS instrument spectral range and spectral sampling are tuned to identify the key spectral features observable in the martian atmosphere, namely water ice, water vapor, dust and CO_2_. The design of the instrument interferometer is largely unchanged from OTES, though the CVD beamsplitter was enlarged to accommodate the larger telescope size. The same antireflection microstructure/beamsplitter approach was used as on OTES and the laser diode/photodiode mounting was tied to the beamsplitter housing to enable a more reliable laser metrology assembly alignment. Otherwise, the dual voice coil linear motor and outrigger assembly used is an identical design to that which flew on MO and MGS TES. The electronics were redesigned from the OTES instrument in order to improve the onboard processing capabilities (data compression), stepper motor control, complex acquisition strategies and enhanced sampling frequency of the servo/metrology interferometer (a 2x improvement over OTES). The enhanced sampling of the metrology interferometer enables a more robust interference rejection needed to accommodate pointing mirror-induced vibrational disturbances to the servo control and ultimately infrared sampling electronics chain.

The primary goal of the EMIRS instrument is to obtain spectra with low instrument noise, high spectral sampling, and nearly-complete geographic/local time coverage to be able to determine the distribution and concentrations of key atmospheric constituents over the martian day and ultimately seasons. The EMM CONOPS strategy provides opportunities for complete geographic coverage of all times of day in less than 10 days of observations (§7.1). During the primary science phase, EMIRS acquires data of ∼1/2 the martian disk within 1/2 an hour of observing. This observation sequence, which include calibration activities, is carried out ∼20 times per orbit and produces data with surface sampling of better than 300 km/px up to 70° emission angle, with the highest resolution data being ∼100 km diameter pixels. EMIRS will use these data to provide global maps over 8 local time bins (∼3 hours) of the column integrated dust opacity, column integrated ice opacity, water vapor abundance and atmospheric temperature profiles to altitudes of 60 km.

### EMIRS Measurement Requirements

#### EMIRS Science Traceability Matrix

#### L2 Requirements

EMIRS provides measurements that meet EMM mission objectives through two investigations as show in the Science Traceability Matrix (Table 2). Specifically EMIRS contributes to Science Objective 1 (Characterizing the state of the lower atmosphere) and Objective 2 (Providing linkages from the lower atmosphere to the exosphere) (Amiri et al. 2020; Almatroushi et al. 2021, this volume) through Investigation 1 (Determine the three-dimensional thermal state of the lower atmosphere and its diurnal variability on sub-seasonal timescales) and Investigation 2 (Determine the geographic and diurnal distribution of key constituents in the lower atmosphere on sub-seasonal timescales). In order to meet the EMM goals, EMIRS will address four key parameters that drive instrument performance and ultimately instrument design.

**Table 2:** The simplified EMIRS Science Traceability Matrix illustrates linkages and requirement flow from mission-level investigations to instrument requirements. The EMIRS as-built instrument performance is detailed in Table 4

EMM Investigation	Level 1 Science Requirements	Level 2 Requirements				Level 3 Instrument Requirements
		Physical Parameter	Physical Parameter Requirement	Observable Quantity	Observable Quantity Requirement	
Investigation 1	Determine the three-dimensional thermal state of the lower atmosphere and its diurnal variability on sub-seasonal timescales.	Temperature profiles <50 km.	*Vertical Resolution*: 10 km for 0-50 km altitude *Temperature accuracy*: ±2.0 K for 0-25 km altitude ±4.0 K for 25-40 km altitude ±10.0 K for 40-50 km altitude	Absolute radiance of CO_2_ absorption band (7-8 μm and 14-16 μm)	*Diurnal*: In any given span of 10 days, the 8 three-hour intervals defining a complete diurnal cycle are sampled with at least 80% coverage of longitudes in: ≥6 of 8 local time intervals for all latitudes equatorward of ±30°, ≥4 of 8 local time intervals for all latitudes equatorward of ±50°. In any given span of 10 days, at least one of the 8 three-hour intervals defining a complete diurnal cycle is sampled with at least 50% coverage of longitudes for all latitudes equatorward of ±80°. *Geographic*: ≥80% of the geographic area of Mars (regardless of local time) sampled more frequently than every 72 hours. Latitudes equatorward of ±80° sampled more frequently than every 72 hours. *Seasonal*: Observations over 1 full Martian year *Observation Geometry*: Coverage requirements apply to emission angles ≤70°. *Spatial Resolution*: ≤300 km at nadir. *Spectral sampling*: ≤5 cm^−1^ & ≤10 cm^−1^ measurments *Absolute radiometric accuracy*: ±1.5%.	*Instantaneous FOV*: 5.7 ± 0.3 mrad at FWHM *Scan FOV*: ±25° about 0° mirror position ×1.6° *Spectral sampling*: ≤5 cm-1 & ≤10 cm-1 measurments *# of steps per scan*: 10-17 *# of scans*: 13-20 *Duration per observation*: 10-25 min *Acquisition Time*: ≤4 s at 5 cm^−1^ sampling & ≤2 s at 10 cm^−1^ *Absolute radiometric accuracy*: ±1.5% *Noise level*: ≤4.7×10^−8^ W cm^−2^ sr^−1^/cm^−1^ NESR at 5 cm^−1^ *Spectral range*: 7-8 μm & 14-16 μm
		Surface temperature	*Spectral Region*: 7-12 μm *Accuracy*: ±2 K	Absolute radiance over 7-12 μm		*Same as above except the following*: *Spectral range*: 7-12 μm
Investigation 2	Determine the geographic and diurnal distribution of key constituents in the lower atmosphere on sub-seasonal timescales.	Ice column integrated optical depth at 12 μm	*Spectral Location*: 12 μm *Accuracy*: ±5%	Relative radiance of H_2_O ice absorption bands (10-15 μm) with respect to the continuum.	*Same as investigation 1*	*Same as investigation 1, except the following:* *Spectral range:* 10-15 μm
		Dust column integrated optical depth at 9 μm	*Spectral Location*: 9 μm *Accuracy*: ±5%	Relative radiance of dust absorption bands (8-25 μm) with respect to the continuum.		*Same as investigation 1, except the following*: *Spectral range*: 8-25 μm
		H_2_0 vapor column abundance.	*Spectral Region*: 25-40 μm *Accuracy*: ±2 pr- μm	Relative radiance of H_2_O vapor absorption bands (25-40 μm) with respect to the continuum.		*Same as investigation 1, except the following*: *Spectral range*: 25-40 μm

Requirements for instrument performance, including the NESR, spectral coverage, spectral resolution, pixel FOV for EMIRS were informed by the corresponding requirements in the quantities needed to answer the science questions of the Emirates Mars Mission (Amiri et al. 2020, this volume). For example, the angular size (5.7±0.3 mrad) of an EMIRS pixel was chosen so that its projected size on Mars would be no larger than 300 km from the 44,000 km planned apoapsis altitude, which is roughly the size of the spatial grid used by General Circulation Models (GCM) for Mars (e.g., Forget et al. 1999; Millour et al. 2018). Likewise, the spectral range was chosen to satisfy the science requirement to retrieve surface (1300 cm^−1^) and atmospheric temperatures (550–750 cm^−1^), dust (300–500 cm^−1^; 950–1150 cm^−1^) and water ice (750–900 cm^−1^) aerosols, and water vapor (200–350 cm^−1^). The spectral resolution of 5 cm^−1^ was chosen to enable the reliable retrieval of the water vapor column abundance (e.g. Smith 2002) and temperature profiles (e.g., Conrath et al. 2000).

Numerical experiments were performed to determine the NESR level required to meet measurement objectives. For a given atmospheric state (temperature profile, aerosol optical depth, etc.) and NESR level, a Monte Carlo simulation was performed using a forward radiative transfer model (e.g. Smith 2002) to produce thousands of simulated EMIRS spectra. Retrievals for atmospheric temperature, aerosol optical depth, and water vapor column were then performed on each of the simulated spectra. The calculated RMS variation in the retrieved values for each quantity were then used to characterize their uncertainty for the chosen instrumental NESR level and atmospheric state used as input for the simulation. By testing a range of different input NESR values in the simulation over a wide range of expected atmospheric state parameters, the NESR requirement of 4.7x10^−8^ W cm^−2^ sr^−1^ /cm^−1^ in the wavenumber range from 1250 to 357 cm^−1^ in 5 cm^−1^ mode was found to produce sufficiently low uncertainties on all retrieved quantities as listed in Table 3 to meet science requirements.

**Table 3:** Predicted EMIRS Uncertainties for given retrieved quantities from 150-350 K Planck radiances given EMIRS instrument performance (Table 4 and Table 5)

Retrieved Quantity	Parameter Uncertainty
Surface Temperature	<2 K
Atmospheric Temperature (0–25 km)	<2 K
Atmospheric Temperature (25–40 km)	<4 K
Atmospheric Temperature (40–50 km)	<10 K
Aerosol Optical Depth (dust and water ice)	<0.03 or 5%, whichever is larger
Water Vapor Column	<2 precipitable microns

**Table 4:** EMIRS as-built instrument parameters and performance

Parameter	Value
Spectral Range	1666 to 100 cm^−1^, (6-100 μm)
Spectral Sampling	10 cm^−1^ & 5 cm^−1^
Telescope Aperture	17.78 cm
f/#	f/3.3
Instantateous Field of Wiew (FWHM)	5.1 mrad (elevation) × 4.6 mrad (azimuth) 5.5 mrad (85% encircled energy)
Field of View (FWHM)	32.5 mrad (elevation) × 32.5 mrad (azimuth)
Scan Field of View	±60 ° around nadir
Detector	uncooled deuterated L-alanine dope triglicine sulfate (DLaTGS) pyroelectric
Detecotor D* (avg for all elements)	9.4 × 10^8^ cm Hz$^{1/2}$ W^−1^ at 10 Hz, 22 °C
Detector Responsivity	3606.7 V/W @ 10 Hz, 22°C
NESR	<∼2.2 E^−8^ W cm^−2^ sr^−1^/cm^−1^ @ 10 cm^−1^ sampling
Cycle time per measurment	1.8 s plus 0.2 s scan reversal time (10 cm^−1^) 3.6 s plus 0.2 s scan reversal time (5 cm^−1^)
Metrology laser self-apodized wavelength (25°C)	0.846 μm
Michelson mirror travel (max)	±0.686 mm
Michelson mirror velocity	0.26 ± 0.013 mm/sec
Sampling Frequency	625 Hz
Number of bits per pixel	16
Number of samples per interferogram	2220±20 (5 cm^−1^) 1120±10 (10 cm^−1^)
Nominal data volume per collect (2 or 4 seconds)	Housekeeping: 160 bytes Science: 25,874 bytes (9 packets)
In Flight Calibration	2 point calibration, internal v-groove target & spacelooks
Calibration Target Emissivity	0.98±0.005
Thermal requirments	Performance in Specification: +10 °C to +40 °C Allowable Flight Operational Range: -10 °C to +45 °C Nonoperational proto-flight survival range: -20°C to +55°C
Solar protection	Autonomous pointing mirror stowed
Mass	14.715 kg (including interface adapter plate)
Power	Average Operational: 22.24 W Standby: 18.6 W Operational Heater: 5.7 W
Dimensions	52.91 cm × 37.54 cm × 34.57 cm

### EMIRS Design

#### Opto/Mechanical

The EMIRS optical system (Fig. 5) uses a compact Cassegrain telescope design with a 17.78 cm diameter f/3.3 Ritchey-Chretien primary telescope, which is fed by a 45° flat rotating mirror that enables single axis pointing. Following the primary mirror, the light is fed to a 45°fold mirror that changes the plane of the beam. This is followed by an off-axis parabola that converts the telescope output beam to a near-collimated optical beam with an afocal ratio of 10. This beam is then directed into the Michelson interferometer beamsplitter held by a radial 3-point flexure mount. The moving mirror is mounted on a dual voice-coil linear motor assembly with ±0.578 mm of travel and the fixed mirror held in a radial 3-point flexure mount (Fig. 7). The entire interferometer is mounted to a precision machined honeycomb, dual skin plate that is mounted and vibration isolated by 3 grommets and O-ring snubbers. This design is heritage from TES (Christensen et al. 2001) and OTES (Christensen et al. 2018). The two separate beams are recombined at the beamsplitter and are then sent to the off-axis parabolic imaging mirror that focuses the modulated light onto the detector array. Each of the 9 detectors is canted at an angle to match the incoming rays and is fitted with a chemical vapor deposited (CVD) diamond lens that is pressure bonded to the outside of the custom TO-18 (a common electronics component package) detector housing.

**Fig. 4:**
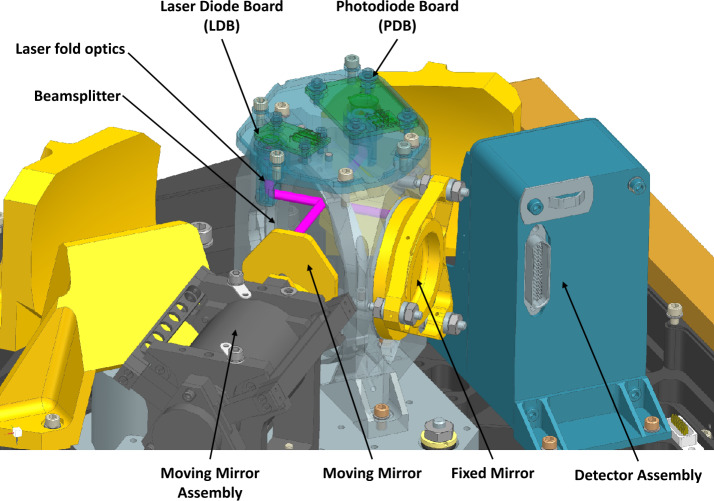
Metrology sub-assembly view. The path of the EMIRS metrology laser is presented in pink and key components are labeled

As with OTES, the EMIRS beamsplitter is a CVD diamond substrate, though the EMIRS beamsplitter is significantly larger 60 mm in diameter than the 38 mm diameter OTES beamsplitter (Christensen et al. 2018) but remains 1 mm thick. The diamond beamsplitter substrate was fabricated by Diamond Materials. The beamsplitter is bonded into a retaining ring via radial epoxy bonds that has 3 flexure mounts which are secured to the beamsplitter housing. This design, functionally unchanged from OTES, aside from the increased diameter, provides precision alignment over the instrument operational temperatures, accommodates the low thermal expansion and high conductivity of diamond, and is mechanically robust to launch loads. The diamond was etched with a antireflection microstructure (ARM) by TelAztec that significantly improves the overall system throughput as first surface reflections due to the high index of refraction diamond would significantly degrade system performance if not included (Christensen et al. 2018). On the opposite side of the ARM, the diamond substrate is coated with a germanium beamsplitter coating tuned to reflect and transmit 50% of the energy. Both the ARM and the germanium are applied to a portion of the beamsplitter, leaving a chord across the top where the metrology laser (VCSEL laser diodes, with a nominal wavelength of 850 nm) coatings, antireflective and beamsplitter are applied. This ensures that the beamsplitter geometry functions the same for both the laser metrology and infrared signals. Further the metrology laser also has a portion of the fixed and moving mirrors to ensure that the laser metrology and infrared signals are co-aligned. The metrology assembly (Fig. 4) provides fringe counting and velocity control to the servo and is located on top of the beamsplitter in a small housing, in a design change from OTES (Christensen et al. 2018) where two metrology lasers were slightly off axis. For the EMIRS design, the single VSCEL laser is on-axis, and in order to protect the photodiode from direct self-illumination, uses a combination of a retro reflector and quarter wave plate to change the polarization of the outgoing (and incoming) laser radiation (Fig. 4). This design permits highly controllable alignment of the metrology system and provides additional margin for any misalignment that may occur due to launch loads or temperature variations.

**Fig. 5:**
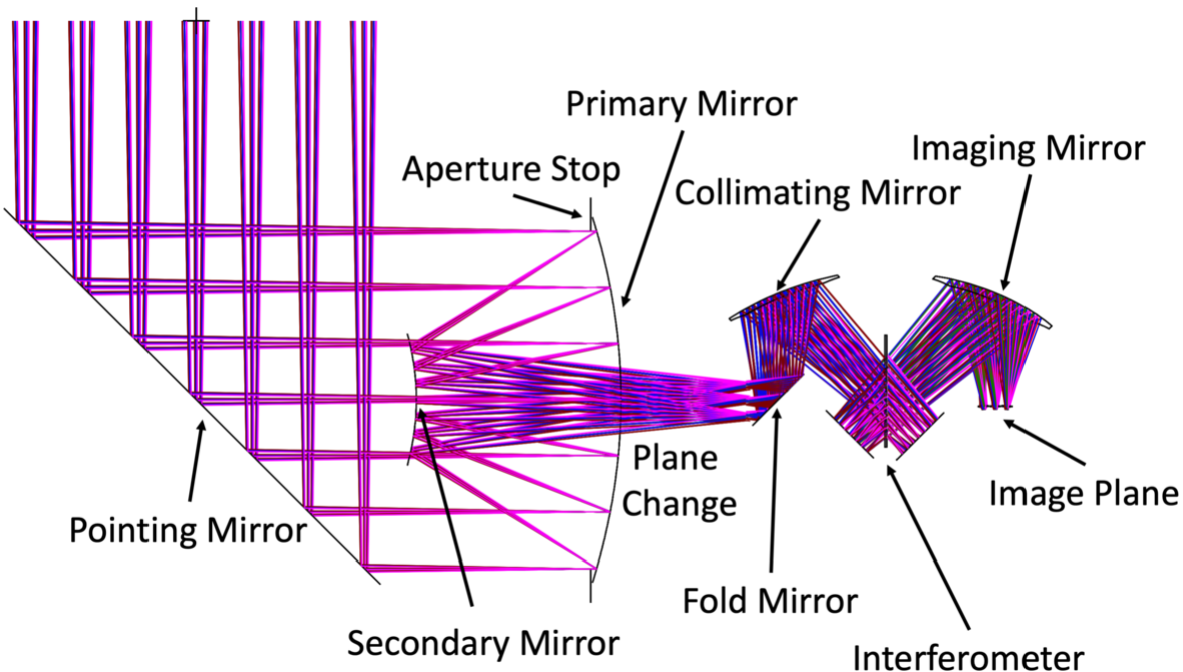
Unfolded EMIRS optical design where the two planes of the optical system are shown (left) telescope optical design, (right) aft optics plate optical design. Pointing mirror not pictured

EMIRS is primarily aluminum in construction with strategic isogrid to improve the mechanical performance to meet launch and pyro shock loads. Due to a mid-development change of the S/C instrument panel to a composite panel, the rigid mount to EMIRS was adapted using a S/C provide adapter plate. EMIRS directly mounts to the aluminum adapter plate which uses titanium flexures to mount to the S/C instrument panel. The EMIRS screws are injection bonded with epoxy after being secured to the plate to remove any residual gaps in the through holes, fixing the EMIRS alignment to the feet of the adapter plate, which is mounted via 3 precision alignment pins and 8-high strength screws to the instrument panel inserts. The EMIRS rotary mirror has internal baffles, as does the secondary mirror and the primary mirror to help reject stray light. These baffles and the internal instrument cavities are painted with a diffuse Aeroglaze Z306 paint (∼100-200 μm thick) which has a high emissivity across infrared wavelengths to further aid in the rejection of stray light (e.g., Adibekyan et al. 2017). While the emissivity has been shown to vary by from 0.60-0.95 over the thicknesses applied at long wavelengths (e.g., >25 μm, Adibekyan et al. 2017), as the instrument relies on a full aperture calibration that includes all optical components, these effects are dramatically minimized. Stray light analysis and baffle design was carried out through the use of FRED optical engineering software. The v-groove calibration target is also made of aluminum and is isolated via standoffs from the instrument chassis. This target is painted with PTI PT401, a specular reflective paint, that enables multiple bounces within the v-groove, further improving the emissivity response to near unity across the EMIRS measurement wavelength range.

#### Interferometer

As with previous ASU-built instruments, EMIRS uses a linear moving mirror to collect double-sided interferograms with the ZPD centered in the range of travel (Fig. 3) in a Michelson interferometer configuration. The 0.846 μm laser metrology interferometer system uses the same (Fig. 4) beamsplitter (with a different coating) and the same moving mirror and fixed mirror in order to simplify internal alignment. On every zero crossing of the laser metrology signal, the infrared chain is sampled in order to provide an even physical spacing of the infrared signal. Due to spacecraft vibrations and vibrations induced by the EMIRS pointing mirror the zero-crossing position is predicted via fits to the sinusoidal signal and the predicted position vs actual position is also used to control the mirror velocity. The EMIRS instrument acquires infrared data that have 2230 ± 20 samples in 4 second, 5 cm^−1^ mode and roughly half number of samples that in 2 second, 5 cm^−1^ mode. These data are zero-filled to 2230 and 1115 respectively prior to performing the discrete Fourier transform (DFT). This zero filling ensures a constant spectral sampling regardless of the exact number of infrared data points acquired by the instrument. The laser wavelength was determined by fitting the infrared spectral data to a known target/atmospheric features such as CO_2_ and H_2_O vapor. EMIRS packetizes these interferograms and compresses the data field in the individual packets (§3.3.4) which are then transmitted to the ground. These interferograms are converted to spectra using the DFT with phase correction done using the signed Mertz method (Forman et al. 1966). This approach, excluding compression, is the same approach that was developed for the OTES instrument (Christensen et al. 2018).

#### Detectors

The EMIRS instrument uses a 3x3 array of uncooled deuterated L-alanine doped triglycine sulfate (DLaTGS) pyroelectric detectors fabricated by Leonardo-MW Limited, formerly Selex-Galileo. OTES used detectors manufactured by the same company though the EMIRS detectors are housed in a TO-18 electronics package and have a detector element 1.6 mm in diameter. These detectors are packaged in a mount that creates a small angular offset to match the angle of the incoming rays with the detector plane through the 2.8-mm diameter CVD diamond lens. The EMIRS detectors have an average Specific Detectivity (D*) of $9.4 \times 10^{8}$ cm Hz$^{1/2}$ W^−1^ at 10 Hz, 22 °C and a range of $8.8 \times 10^{8}$ to $1.08 \times 10^{9}$ cm Hz$^{1/2}$ W^−1^ at 10 Hz, 22 °C. The detectors also have an average Responsivity of 3606.7 V/W at 10 Hz, 22 °C and a range of 3268 V/W to 4192 V/W. All performance metrics are provided with the diamond lens installed. In order to ensure the detector remains properly poled, a bias voltage is applied to the internal Field Effect Transistor (FET). Similar detectors were used on the TES, Mini-TES and OTES instruments in various forms. In order to read out the detector with the lowest noise possible, current sources with noise less than that of the detector Noise Equivalent Power (NEP) are used to drive the preamplifier circuit on an independent detector board. This board also provides front-end filtering and AC-couples the detector output so as to block any other high-frequency interference, such as that caused by the h-bridge motor driver for the dual voice coil linear interferometer motor and the pointing mirror stepper motor. In practice, the on-axis detector (detector 5) exceeds performance requirements over the entire wavelength range, while the remaining detectors do not. Detectors 2, 4, 6, and 8 meet performance requirements over the longer wavelength ranges of the instrument. As seen in §6, EMIRS meets the needed coverage and science requirements with the single, on axis detector. In practice, the downlinked detectors can be selected to minimize returned data volume, while meeting the science requirements.

#### Electronics

**Fig. 6:**
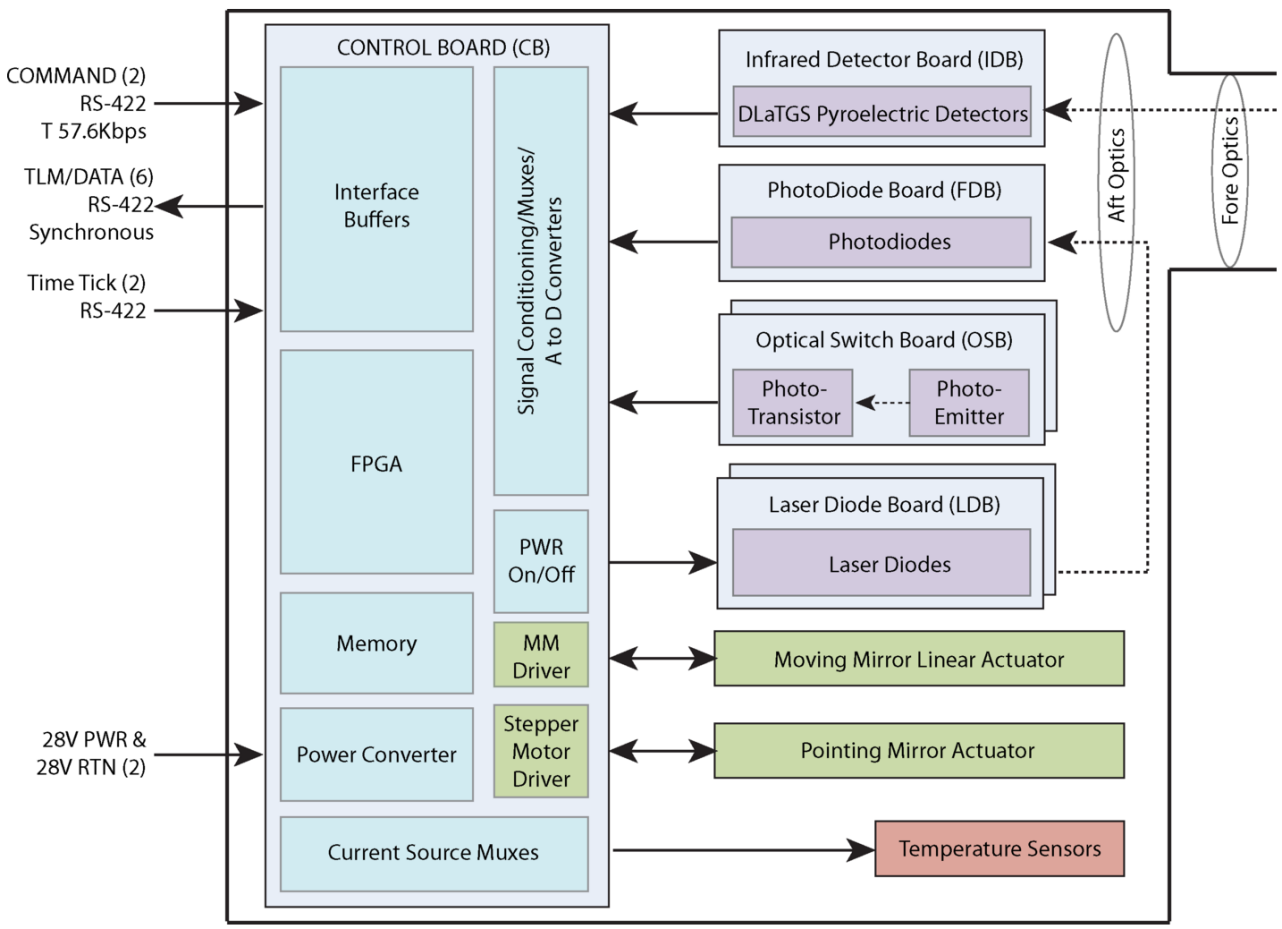
The EMIRS high-level block diagram showing the key functional elements and the instrument to spacecraft interfaces

The EMIRS electronics were designed by Arizona Space Technologies and incorporate a Xilinx Vertex V Field Programmable Gate Array with a LEON 3 floating point processor. These electronics, which are encased in the base of the instrument chassis, provide key instrument functionality. Specifically, the instrument electronics provide S/C power conditioning via an Interpoint DC/DC converter, analog signal processing, digital signal processing, command and data handling, packetization of science and housekeeping packets and control of both the 3-phase stepper motor and the linear voice coil motor drive and tachometer (Fig. 6). EMIRS has 12 electronics boards in total, which are detailed below.

The instrument Controller Board (CB) and Motor Driver Board (MDB) contains roughly 95% of the electronics components, including the power conditioning that accepts 33 V (26-34 V) unregulated S/C power from the redundant S/C power switches and converts it to the necessary internal voltages (±12, ±5, +3.3, ±2.5, ±1) for EMIRS along with other point of load and linear regulators. In addition to providing multiple packetized RS-422 and LVDS data interfaces to the S/C, the CB include the digital servo loop processing that provides the motion control and timing for the sampling of the infrared data chain. The controller board Xilinx Virtex V and LEON 3 processor controls the software commands (though low-level FPGA hardware-based commands to switch physical sensors exist) and the timing of the command execution. It orchestrates the pointing mirror movement, data collection start, timing of commands, and the sampling of all internal hardware interfaces. It also completes “sun in the field-of-view” checking and implements internal safing mechanisms if the angular threshold (11.35° half angle) between the boresight and the sun are violated.

The electronics also respond to spacecraft safe events, ensuring the pointing mirror is closed before power is removed from the instrument. The electronics for EMIRS were completely re-architected from previous instruments to enable enhanced noise rejection of the servo, add pointing mirror control, include onboard lossless compression, the time synchronized readout of multiple detectors and sophisticated command handling.

A significant upgrade to EMIRS is the robustness of the servo control and data sampling chain. More specifically, the digital servo control loop metrology assembly (metrology laser on the Laser Diode Board (LDB) and photodiode on the Photodiode Board (PDB)) are sampled at 40 kHz, double the rate of OTES. These data are used in combination with the dual-voice coil linear motor drive and tachometer to provide a rate estimate that predicts the movement of the moving-mirror, linear-motor system at 5KHz. This rate estimator is key to rejecting vibrational noise. The rate estimator algorithm also predicts the zero crossings for the laser metrology assembly and triggers the sampling of the infrared detector signals, while compensating for the phase delay of the Bessel filter in the Detector electronics boards (the Detector Board and the Detector Daughter Board). Once these data are sampled, they are processed through the onboard Deflate compression algorithm and moved into an FPGA memory location and are sent to the spacecraft via the LVDS interface. The time code in the housekeeping packet (generated on the turn-around of the linear motor) is locked into all science packets in order to assist in the correlation of state of health/housekeeping data to science data.

**Fig. 7:**
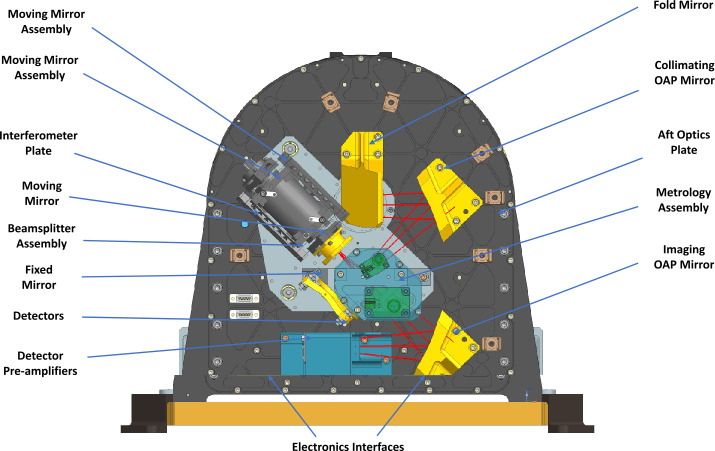
EMIRS “Aft Optics Plate” where the critical optical components of the interferometer are mounted and aligned. The Interferometer assembly (beamsplitter, moving mirror and fixed mirror) are mounted to a vibration isolated plate to maintain precision internal alignment

An optical switch (Optical Switch Board, OSBs) near the center of travel (consisting of 2 sets of redundant photodiodes and light emitting diodes) is used to provide an absolute reference for the interferometer zero path difference (ZPD) where the distance light travels in the two legs of the interferometer is equal. The servo control loop uses this optical switch to ensure that the ZPD is near the center of the range of travel and infrared data sampling of the interferogram. The offset of the ZPD relative to the optical switches on the OSB is set for both scan modes (2 and 4 second) and can be tuned to ensure that the ZPD is always near the center of mechanical travel without physically moving the OSB. During ground testing a “gravity compensation” mode is enabled which provides a constant bias in the position of linear motor as the dual voice-coil, infinite life flexure assembly (Fig. 7) is designed to operate in 0-g.

The same OSBs used on the linear motor are used to determine the absolute position of the pointing mirror. An OSB located on the output of the pointing mirror baffle identifies the coarse location of the pointing mirror while an OSB located on the output of the 3-phase stepper motor shaft is the fine-location sensor and can locate the pointing mirror uniquely to a single step (0.03°). The EMIRS electronics use this knowledge to power on the instrument safely, avoiding sun in the field of view, and uniquely identify the home position.

The control of the linear motor and pointing mirror motor are delegated to the MDB to avoid any high-frequency noise on the CB. The MDB contains independent H-bridge drive electronics that drive the infinite life, dual voice-coil linear motor (BEI Kimco) and the 3-phase stepper motor (Avior Controls). The dual voice-coil linear motor and outrigger assembly are of identical design to that of TES which operated non-stop for ∼10 years at a 1/2 Hz cycle, completing well over 150 million actuations over its operational life. A qualification unit for the 3-phase stepper motor was life tested over operational temperatures (-20 to 50 °C), over voltage ranges (26 to 35 V), in vacuum to 2.6x the operational design life for nominal EMIRS science activities. Upon disassembly of this qualification unit, no degradation or wear in components or lubricant was observed for the 571 million steps actuated.

EMIRS electronics implement 3 fault protection watchdogs. The first is a processor watchdog which looks for latch-ups and other internal faults, the second is a spacecraft status message watchdog which evaluates the health and safety of the instrument in-response to certain spacecraft issues (e.g. 2 missing messages or indication of safe mode). Finally, a sun-vector watch dog computes the angle of the EMIRS boresight to the position of the sun and evaluates this against a keep out zone (11.35°). While the pointing mirror is moving this computation occurs at 10 Hz and when the pointing mirror is away from its stowed position (beyond 54° from its internal home reference, or 126° from nadir) the computation occurs at 2 Hz.

The EMIRS firmware is non-changeable in flight, but the flight software, stored in non-volatile memory, can be updated via patch or complete image upload into one of the 4 flight software boot locations. Temporary changes to all key parameters can be made via parameter update style commands which can modify the more than 20 servo parameters, the movement profile of the pointing mirror, various internal thresholds/instrument parameters, etc. These changes are lost on power off or reset of the processor, triggered either by an external reset command or a processor watchdog.

#### Thermal Design

The EMIRS instrument requires thermal stability of <0.1°C per minute in order to acquire well-calibrated data. The driver of this stability requirement is the duration between internal calibration observations that bracket the nominal science data acquisition, 20-30 minutes apart. However, space-looks that provide the second point of the 2-point full aperture calibration are used to trend the instrument temperature/radiance are acquired at the beginning and end of every scan row, typically with no observations of Mars being further than 2 minutes from a space observation apart. The instrument thermal stability is achieved by the conductively isolated EMIRS interface mounting plate, preventing fast transients. However, the bottom of this interface plate (which has a large cutout in the middle (Fig. 3) and the EMIRS instrument bottom are high emissivity and thus radiatively coupled to the instrument deck which provides a long-term thermal sink. The instrument also has a set of operational heaters (∼5.6 W) on the EMIRS interface plate which are thermostatically protected (to protect the instrument from overtemperature beyond ∼43 °C) though in practice they operate at 100% duty cycle elevating the temperature of EMIRS by roughly 5-7 °C higher than would be achieved without these operational heaters. To further protect EMIRS there are redundant survival heaters with a cut-on at -8 °C and cut off at -2 °C mounted directly to the instrument chassis sides (Fig. 3). The EMIRS instrument will operate within specification from +10 to +40 °C, out of specification (reduced performance from -10 to +45 °C and can survive temperatures from -20 to +55 °C measured at the detector housing. If exposed to temperatures from +65 °C to ∼+80 °C the DLaTGS detectors must undergo a period of re-poling where the detectors will remain unpowered for roughly 48 hours, following this excursion the instrument can return to normal operations with minimal to no performance impact. If exposed to temperatures >+80 °C, the detector DLaTGS elements may be permanently damaged. This exposure limit is the driver for the majority of the sun-in-the-field-of-view protection as analysis shows that if sun is directly viewed by the instrument at Mars, the detector would exceed the 80 °C limit in <0.005 seconds with conservative assumptions about heat dissipation in the detector element.

#### S/C Interfaces

The EMIRS instrument has three communications interfaces, namely 2 synchronous RS-422 interfaces for housekeeping and commanding and one high-speed LVDS interface for science data (Fig. 6). All packets are encoded in the CCSDS protocol which includes internal checksum validation in order to ensure reliability of the interfaces. The CB accepts instrument commands from the spacecraft via RS-422 and stores them in a command queue which executes sequentially. Additional spacecraft messages, namely the status message (2 Hz) that provides the spacecraft state and sun position relative to the spacecraft coordinate frame and time update message (1 Hz) that contains the spacecraft clock information are delivered on this same interface. An RS-422 interface provides the time-update pulse per second which allows EMIRS to lock/synchronize the spacecraft time into its internal clock. The instrument plays back CCSDS packetized housekeeping/state of health data which contains numerous data types/telemetry points (detailed in the EMIRS Instrument Data User’s Guide provided with public data releases, §7) via RS-422 interface. Compressed, packetized science data is played back via a high speed LVDS interface. Prior to sending science data via the LVDS interface, the EMIRS instrument acquires 16-bit interferogram data, optionally compresses this data field using the Deflate algorithm on active detectors, selected via a detector mask. Once the data have been processed packets for active detectors are populated while packets for inactive detectors leave the data field blank. These data are then sent across the LVDS interface.

#### EMIRS Radiation and Contamination Mitigation

EMIRS radiation compliance is accomplished through parts selection and estimates of radiation levels as provided by the aluminum shielding of the instrument chassis. EMIRS instrument was designed to survive 20 krad environments. The detector DLaTGS element does not have a guaranteed radiation specification, though the same composition detector worked on MGS TES for >10 years, >5 years for Mini-TES and >4 years for OTES. These radiation environments are similar to what will be experienced by EMIRS. Over the 10-year life span of MGS TES it is estimated that the 20 krad radiation dose was exceeded and the instrument detectors showed no signs of degradation. EMIRS was built following the EMM project Contamination Control Plan to minimize any organic materials carried on the instrument. Given the operational wavelengths of the instrument (6-100 μm) contamination of the optical surfaces would have to be extreme (> μm, uniform coatings) to degrade the performance of the instrument. This is based on the extremely dusty environments experienced by the Mini-TES instruments (Ruff et al. 2011) with thick, relatively uniform dust deposition on the primary mirror. During assembly, integration and test, EMIRS was kept in an environment bag while in storage that was purged with dry LN_2_ boil off, until the instrument enclosure was complete enough to provide a direct purge to the instrument cavity. This purge was carried through the entire duration of the spacecraft integration, transport and launch with minimal outages during purge source changes, and directly prior to launch. Unlike TES, no components in the EMIRS system are hygroscopic, so the purge is primarily to prevent particulate contamination. During assembly witness samples and monitoring of the cleanroom quality at ASU were used to demonstrate that EMIRS was delivered with a cleanliness level equivalent to level D per NASA CR 4740.

#### EMIRS Operational Modes

**Fig. 8:**
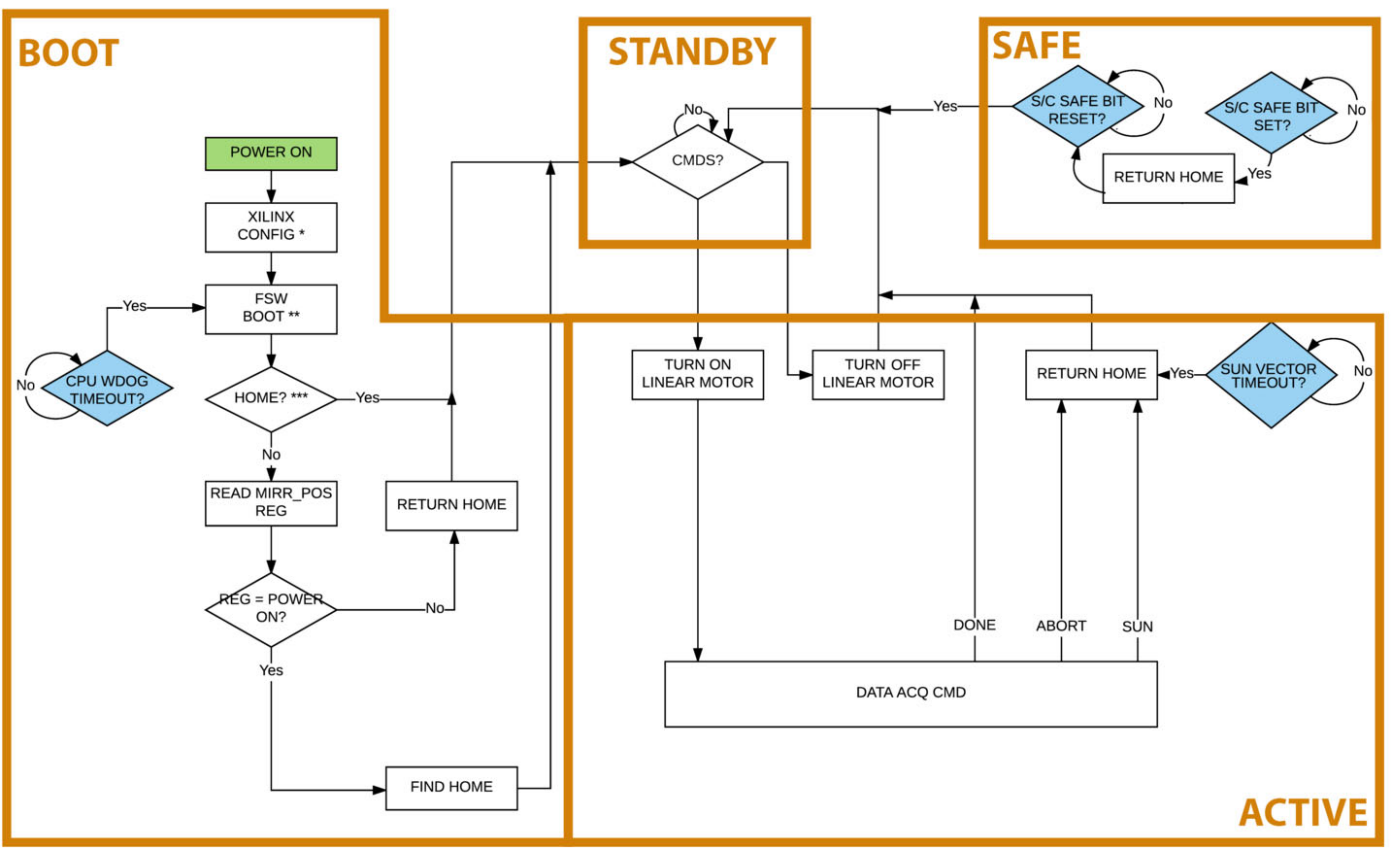
EMIRS modes diagram that illustrates transitions from one mode to another. The EMIRS linear motor must be commanded by the S/C into the off/on configuration

The EMIRS instrument has four primary modes in which the instrument is operated (Fig. 8).

Upon application of power to the instrument, it enters the “Boot” mode, where the FPGA is configured, the flight software is booted and the instrument searches for the pointing mirror home location. This entire sequence takes approximately 7 seconds, and the instrument outputs its first telemetry packet within 8 seconds of power on. The CPU watchdog is also started to monitor the FSW state for latchup. After these steps are successfully completed, the instrument enters “Standby” mode where it awaits software commands and remains in this state unless commanded otherwise. In this state the interferometer motor is typically not powered. If the S/C status message does not arrive for two consecutive packets, or if the S/C issues a “Go-Safe” in the status message, EMIRS transitions to the “Safe” state, where the pointing mirror is rotated to the home position (if not already there). If the status message is missing, EMIRS rejects commands that would move the pointing mirror away from the home position. If the S/C issues the “Go-Safe’ in the status message, EMIRS will reject all commands. The final state is the “Active” state, where EMIRS is actively acquiring data. In this state, any time the pointing mirror is >54° from the home position, EMIRS computes a sun to instrument boresight angle, which if the threshold is triggered, would result in the current acquisition command being aborted and a return to home, and a return to “Standby”. If the command is successfully completed or aborted the instrument also returns to “Standby” where it will accept the next command.

#### Commanding

EMIRS commanding relies on 3 primary commands: 1) EMIRS_ACQ 2) EMIRS_MIRR_ROTATE and 3) EMIRS_LINEAR_MOTOR commands. These commands are implemented in a rule-based planning software entitled OASIS-PS to plan out the command sequence to carry out an observation set. All commands are issued by the onboard S/C sequence engine at the commanded time. If the command relies on the linear motor position (e.g. EMIRS_ACQ) then the command executes on the next linear motor turn around (a 2 or 4 second boundary).

First, the instrument is configured for the specific instrument spectral sampling (10 cm^−1^, 2 second scans or 5 cm^-1,^ 4 second scans) and detector selection (1, 5, 9 or any other combination). This is accomplished via several parameter load commands which update the instrument parameters until next power off or reset. The EMIRS_LIN_MOTOR is enabled 15 minutes prior to the start of an acquisition, which begins the thermal equilibration process as the added power (∼3 W) from the EMIRS linear motor is housed entirely in the aft-optics cavity (Fig. 3). This is followed by an EMIRS_ACQ command, that issues a set of 20 dwells on the internal calibration target. An absolutely timed mirror move command, brings the EMIRS mirror to the start position of the raster scan and 15-30 seconds (depending on the distance traveled) is given for the system to remove any disturbances in the servo as a result of the large mirror movement. Another EMIRS_ACQ command with a given raster size and step increment is issued and the EMIRS instrument autonomously creates the full raster in combination with the S/C slew. Following the completion of this, the EMIRS_MIRR_ROTATE command is issued to return the EMIRS pointing mirror to the internal calibration target. And finally the same internal calibration EMIRS_ACQ command is issued. This is followed by a EMIRS_LIN_MOTOR disable command. The command syntax is as follows:

EMIRS_LIN_MOTOR *enable*/*disable*

*enable* = *power on the EMIRS interferometer linear motor*

*disable* = *power off the EMIRS interferometer linear motor*

EMIRS_MIRR_ROTATE *steps* *direction*

*steps* = the number of steps to rotate the mirror in 0.03° increments (12000 steps per 360° revolution

*direction* = rotate the mirror in the clockwise or counterclockwise direction

EMIRS_ACQ *cal_dwells start_angle dwells step_size steps cols pause*

*cal_dwells* = number of calibration dwells to acquire before and after raster scan

*start_angle* = mirror start position (integer number of steps)

*dwells* = number of dwells/ICKS per mirror position (typically 1)

*step_size* = step size between locations (integer number of 0.03° steps)

*steps* = number of positions per column

*cols* = number of columns per image raster

*pause* = number of ICKS to pause at the start of each row

The EMIRS_ACQ command is capable of carrying out internal calibration activities, though for timing constraints on the EMM S/C and permitting for servo convergence and mirror settling time the command set describe above uses a special EMIRS_ACQ case to both exclude internal calibration target acquisitions from the raster, but also to command internal calibration target acquisitions, via a 0 step size movement.

## Calibration Methodology

Michelson interferometer-based instruments use constructive and deconstructive interference to measure a spectrum in Fourier space. Specifically, a two arm Michelson interferometer uses a beamsplitter to send half of the energy down a fixed length path where it reflects off a mirror, and the other half of the energy is sent down a variable path length where it reflects off a moving mirror. The light reflected of these two mirrors is recombined at the beamsplitter, where half of the energy is sent to the detector and the other half is returned out the entrance aperture (Christensen et al. 2018). At the zero path difference (where the path length of the two arms are equal), the light sent to the detector is perfectly in phase with constructive interference, producing a large signal at the detector, while the light leaving the telescope aperture is 180° out of phase and is perfectly deconstructive interference. Given the energy emitted from the detector follows the opposite path (180° out of phase from the scene energy) with some energy being reflected back to the detector and some energy leaving the optical system, at ZPD, there is no signal from the detector that can be sensed by the detector (Christensen et al. 2018). However, when the scene-based light is out of phase/deconstructive, the light emitted from the detector is in-phase and constructive, thus when the modulated energy from the scene is lowest, the modulated energy from the detector is highest.

Of particular advantage of interferometric systems that use pyroelectric detectors is that only modulated light is measured. Therefore, other potential signals such as warm instrument components that may be radiating on to the detector, do not produce an interferometric signal. Since pyroelectric DLaTGS detectors only produce an electrical signal when the incident energy is changing levels, these would be sources of error simply manifest themselves as a DC offset term and are not measured and do not need to be considered in the instrument calibration (other than the change in temperature the environment may impart on the detector itself).

The EMIRS radiometric calibration is based on a parametric model that describes the instrument performance and can also be used to characterize the absolute radiometric response, or accuracy, of the EMIRS. This model calculates the absolute radiance detected by the EMIRS based on a given set of parameters. The EMIRS calibration uses two calibration measurements: periodic observations of space and the internal calibration v-groove blackbody target. The observations of the internal v-groove calibration target occur at the beginning and end of each observation sequence and space looks occur as a part of the nominal observation sequence as the raster scan is oversized by ∼1° on each side of the martian disk.

EMIRS measures the difference between the external spectral radiance (in W cm^−2^ sr^−1^/cm^−1^) falling onto the detector, and that emitted outward from the detector itself. The external radiance onto the detector is the sum of the radiance coming from the scene through the aperture as limited by the field stop ($R_{\mathit{scene}}$), the radiance from the optics ($R_{\mathit{optics}}$), and any radiance from the field stop and the interior of the instrument that has been modulated through the interferometer. The difference between this modulated external radiance and that from the detector ($R_{\mathit{detector}}$), each weighted by their appropriate viewing solid angle, is the signal measured by the detector.

The electrical signal ($V$) produced by the interferometer is given by:

1 $$ V_{\mathit{scene}} = \left ( R_{\mathit{scene}} - R_{\mathit{detector}} \right ) \cdot \mathit{IRF} $$

where:

$V_{\mathit{scene}}$ = Voltage measurement when viewing the scene

$R_{\mathit{scene}}$ = Radiance of the scene

$R_{\mathit{detector}}$ = Radiance of the detector

$\mathit{IRF}$ = Instrument response function (V/W) – optical power to electrical voltage

The accurate determination of the desired scene radiance ($R_{\mathit{scene}}$) requires knowledge of the two unknown terms in this equation, ($R_{\mathit{scene}} - R_{\mathit{detector}}$) and *IRF*, which in turn requires the observation of two calibration targets of known radiance, both of which are viewed through the identical optical path as the scene (Christensen et al. 2018). In the case of EMIRS, a full-aperture calibration is relatively simple as EMIRS can use its pointing mirror to observe through all optical components, a precision calibration target and space. For OTES, which relies on a small calibration flag behind the primary optics, the calibration equations then rely on the characteristics and temperatures of the fore-optics. For EMIRS, any degradation or uncertainties in temperatures/spectral performance, of these optics is removed without solving any additional equations or relying on any additional temperature measurements.

The measured voltage spectra, $V$, from each observation at each target are given by:

2a $$\begin{aligned} &V_{\mathit{scene}} = \left ( R_{\mathit{scene}} - R_{\mathit{detector}} \right ) \cdot \mathit{IRF} \end{aligned}$$

2b $$\begin{aligned} &V_{\mathit{space}} = \left ( R_{\mathit{space}} - R_{\mathit{detector}} \right ) \cdot \mathit{IRF} \end{aligned}$$

2c $$\begin{aligned} &V_{\mathit{cal}} = \left ( R_{\mathit{cal}} - R_{\mathit{detector}} \right ) \cdot \mathit{IRF} \end{aligned}$$

where:

$V_{\mathit{space}}$ = Voltage measurement when viewing space

$R_{\mathit{space}}$ = Radiance of space

$V_{\mathit{cal}}$ = Voltage measurement when viewing the internal calibration target

$R_{\mathit{cal}}$ = Radiance of the internal calibration target

Using these measurements, the instrument response function ($f$) is be computed using the space and the internal calibration target spectra:

3 $$ \mathit{IRF} = \frac{\left ( V_{\mathit{cal}} - V_{\mathit{space}} \right )}{\left ( R_{\mathit{cal}} - R_{\mathit{space}} \right )} $$

Thus, when substituting values appropriately, the EMIRS absolute spectral radiance equation for the scene is:

4 $$ R_{\mathit{scene}} = \varepsilon _{\mathit{scene}} B_{\mathit{scene}} = \frac{\left ( V_{\mathit{scene}} - V_{\mathit{space}} \right )}{\mathit{IRF}} + R_{\mathit{space}} $$

where:

$\varepsilon _{\mathit{cal}}$ = Emissivity of the calibration target

$\varepsilon _{\mathit{scene}}$ = *Emissivity of the scene*

Expanding Equation (4) and substituting yields the final equation for determining the calibrated radiance of the scene as measured by EMIRS’s full aperture calibration (Christensen et al. 2018).

5 $$ R_{\mathit{scene}} = \varepsilon _{\mathit{scene}} B_{\mathit{scene}} = \left ( \varepsilon _{\mathit{cal}} R_{\mathit{cal}} - \varepsilon _{\mathit{space}} B_{\mathit{space}} \right ) \frac{\left ( V_{\mathit{scene}} - V_{\mathit{space}} \right )}{\left ( V_{\mathit{cal}} - V_{\mathit{space}} \right )} + \varepsilon _{\mathit{space}} B_{\mathit{space}} $$

where:

$B_{\mathit{cal}}$ = Planck radiance of the internal calibration target (RTD measured temperature)

$\varepsilon _{\mathit{cal}}$ = Emissivity of the internal calibration target

$B_{\mathit{space}}$ = Planck radiance of space (2.7 K, cosmic microwave background)

$E_{\mathit{space}}$ = Emissivity of space In practice during the on-orbit calibration process, the EMIRS processing pipeline will determine the nearest space and internal calibration observations to the given scene observation and will use an interpolation scheme to remove any slow thermal drifts. This interpolation scheme evaluates and trends the stability of the instrument via thermistor measured temperatures on the detector assembly and using the multiple space looks in-fills a nearest modeled space and calibration observation to the science observation. This further improves the overall instrument performance and enhances the robustness to long term thermal variations.

### Absolute Calibration

The terms in Eq. (5) and their associated uncertainties determine the absolute performance of the EMIRS instrument. Specifically, the variations in the signal terms (V_scene_, V_space_, and V_cal_) is dictated by the noise level of the instrument and consequently each measurement. Averaging multiple spectra or bands can be performed and reduces the instrument noise by the sqrt($n$) where $n$ is the # of channels or spectra averaged together. This also applies to the sampling methodologies of EMIRS such that EMIRS has a sqrt(2) lower noise when acquiring measurements in 10 cm^−1^ mode as compared to 5 cm^−1^ mode. These measurement errors are characterized in EMIRS thermal vacuum testing where the standard deviation of measurements staring at a fixed target of known temperature with a stable instrument are used to determine the NESR. The details of the instrument performance are provided in §6.

The absolute instrument accuracy is primarily dictated by the knowledge of each the emissivity and temperature of the internal calibration target given the properties of space well known. The temperature uncertainty is dictated by the target uniformity and the precision/accuracy of the RTD temperature sensors mounted to the rear of the v-groove calibration target (Fig. 3). The emissivity is a function of the target geometry and paint (PT-401) and is determined as treating the calibration target as a “scene” term of Eq. (5). In this scenario two precision calibration targets of known temperature and emissivity are used as the reference and the calibration quality of those targets is transferred to the EMIRS internal calibration target and are used to determine its emissivity and any temperature offset from the RTD sensors.

### Test Equipment and Facilities

The EMIRS instrument was assembled tested and calibrated in the Interdisciplinary Science and Technology Building 4 (ISTB4) at Arizona State University. ISTB4 houses two instrument assembly cleanrooms, an ISO Class 7 (class 10,000) with ISO Class 6 (class 1,000) flow benches along with a larger ISO Class 8 (class 100,000) cleanroom that houses a thermal vacuum chamber dedicated to instrument level testing. All test equipment, including power supplies, multimeters etc., are calibrated to NIST standards on a routine basis. All sub-components of EMIRS were tested either at the manufacturer (and verified upon delivery) or tested in house to ensure the components were up to functional and performance specifications. EMIRS assemblies were tested independently in ambient conditions while electronics were tested over workmanship temperature ranges to ensure functionality prior to integration into the system. As described below, following every major subsystem test, instrument move, sub-assembly integration, EMIRS performed numerous characterization tests to prove the functionality of the instrument. The ambient tests that occurred during the EMIRS system integration and test program include: 1) field of view testing for definition and alignment to the EMIRS reference optical cube 2) out-of-field response (encircled energy), 3) wavelength range and spectrometer data sampling, 4) data and power interfaces, 5) modulation efficiency, and 6) orientation testing. At outside facilities the instrument was tested to proto-flight levels for random and sine vibration (Grey’s Engineering in Tempe, Arizona) and underwent Electromagnetic Susceptibility and Compatibility testing (DNB Engineering in Chandler, Arizona). Following ambient testing, the instrument was installed in the ASU Thermal Vacuum Chamber where the instrument gain was verified, the performance (temperature/emissivity) of the internal calibration target was characterized and the instrument response function and radiometric precision/accuracy were verified over temperature in vacuum. Table 5 provides the uncertainties and values associated with the sub-components that control the radiance error of EMIRS. These instrument uncertainties, among other observation conditions, drive the uncertainties of retrieved parameters of the martian atmosphere and surface temperature.

**Table 5:** EMIRS Internal Calibration Target Uncertainty over instrument operational temperatures (10-35°C)

Variable	Nominal value	Uncertainty	Radiance error
Cal Target Temp	25 °C	0.5 °C	0.64%
Cal Target Emissivity	0.99	0.005	0.51%

These equations remain valid if the instrument remains a near constant temperature. Given the EMIRS instrument will drift in temperature this assumption does not hold perfectly true. However, EMIRS observes the internal calibration target within ∼15 minutes of any given observation and obtains a space look within 1-2 minutes of any observation. Further, experience has shown (Christensen et al. 2001, 2018) that DLaTGS detectors are reliably stable and during thus the instrument response function (determined every 15 min or so) will remain constant. In fact, the instrument IRF is likely to remain unchanged over nearly the entire mission at a given temperature. Any changes to the instrument temperature are trended and corrected for using the space looks which permit the determination of the detector radiance on much shorter timescales. However, uncertainties in the detector radiance (temperature) directly translate to uncertainties in the derived radiance and temperature of the scene given it is a simple additive term in Eq. (5).

#### EMIRS Interface & Benchtop Testing

The EMM project provided a spacecraft simulator to support the EMIRS integration and test program. This simulator provided flight like interfaces (RS-422 & LVDS) with proper termination and a command-able, calibrated power supply, along with emergency shutoff. On top of this low-level interface, EMIRS was commanded via custom programmed LabView virtual instruments. These commands were issued to the spacecraft simulator via dedicated TCP/IP protocol connections on a private network. Command sequences were scripted using National Instruments’ TestStand product which enables the robust, repeatable error checking and script execution with built in holds that wait on specific telemetry points. After integration was completed, following every major test conducted, the EMIRS Instrument Functional Test (IFT) was run. The IFT tests all major instrument functions, hardware, and software of the instrument and automatically flags many common issues. The IFT was run 36 times during the system integration and test program. It was often coupled with Comprehensive Performance Tests (CPT, 2 external temperatures) and Limited Performance Tests (LPT, 1 external temperature) that would enable a radiometric characterization of the instrument. For this series of tests, a large aperture (8″) precision, calibration target could be oriented to fill the EMIRS FOV. The EMIRS system integration and test program completed 158 LPTs and 28 CPTs.

#### Thermal Vacuum Testing

**Fig. 9:**
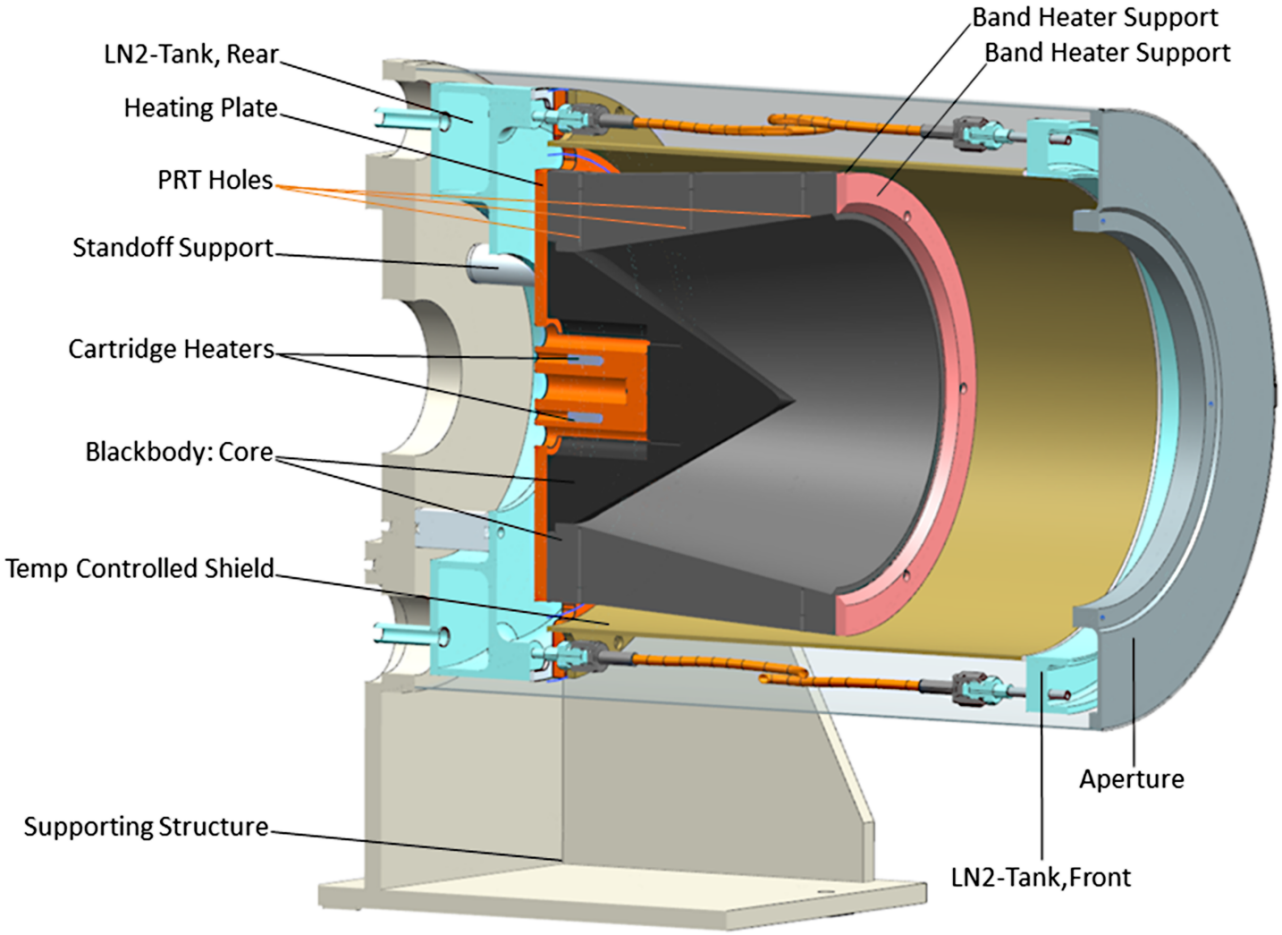
BCU Cross section that illustrates the key components of the inverted cone blackbody. The inverted cone geometry minimizes the overall length without sacrificing performance

The EMIRS thermal vacuum testing serves multiple purposes, most specifically to characterize the instrument behavior over temperature and ensure workmanship functionality. In order to accomplish this, two precision Bench Calibration Units (BCU). units that are precisely controlled to specific temperatures and read out with 18 (each) precision, NIST-calibrated platinum resistance thermistors with uncertainties of ±0.1 °C were used. Due to the large aperture of the EMIRS instrument as compared to previous instruments a new set of inverted cone BCUs (Fig. 9) were created and analyzed for this program. The emissivity of these targets was determined to be 0.99893±0.0002 from 5-100 μm over 7-350 K including the effects of a warm (300 K) instrument radiating onto the blackbody surface. The emissivity was determined based on the spectral properties of the PT-401 paint and the analysis of the geometry of the blackbody (Prokhorov et al. 2009) using the Virial International STEEP 320 software package that conducts Monte Carlo simulations of axial symmetric blackbody radiators. The temperature sensors are assembled directly onto the Blackbody core at approximately 1.25 mm from the emitting surface.

The cartridge heaters were mounted onto a heating plate in order to facilitate an even heat distribution from the rear part of the unit. In order to reduce the temperature gradient along the length of the unit, a polyamide band heater was precisely sized and located mounted on an aluminum band heater support at the front part of the unit. The cooling part is provided through two liquid nitrogen tanks located at front and rear part of the unit. These two tanks are mechanically attached through an aluminum shield, providing a uniform environmental temperature along the blackbody unit. The main unit is isolated by stainless steel washers and standoff supports.

The instrument was fitted with 14 instrument-monitored RTDs to correlate the instrument response to the thermal environment. Four of these NIST calibrated flight thermistors are mounted to the underside of the internal v-groove calibration target and are evenly spaced so they measure the same proportional areas and can be easily averaged without a weighting function to capture the average calibration target temperature. This calibration target is viewed through the full aperture and optics, providing accurate calibration (§4).

In thermal vacuum testing, the same GSE as described above was used to measure the instrument performance via chamber feedthroughs. Additional ASU TVAC related chamber related GSE was used to control the components of the chamber, including the platen, shrouds, calibration targets, and instrument plate heaters. The majority of these devices are controlled by automated controllers, heaters and valves that permit the chamber to run efficiently with minimal user input. The chamber is instrumented with >30 Resistance Temperature Detectors (RTDs) and the precision calibrated blackbodies are monitored with 30 redundant PRTs each. All of these temperatures are monitored by the chamber GSE, which are then delivered to the EMIRS GSE (and ultimately database) by shared network variables in LabView. This enables the EMIRS GSE to receive these critical telemetry points and store them in the EMIRS System Integration and Test database, with co-incident time-tagged data for additional processing. Again, NI TestStand was used to automate the EMIRS TVAC, with over 500 TVAC tests being conducted, requiring roughly 60 minutes each to complete.

**Fig. 10:**
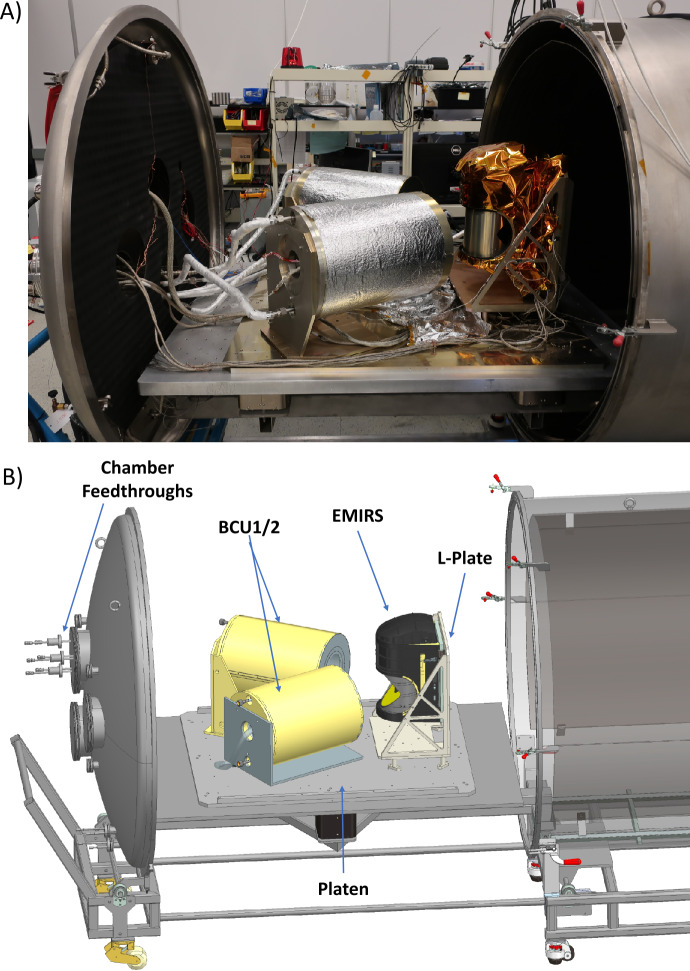
(a) EMIRS fully blanketed and mounted onto the “L-plate” which positions the telescope directly at the level of the BCU apertures. (b) mechanical model of the TVAC configuration with components labeled

In order to drive the instrument to the necessary temperature ranges while approximating the radiative environment of space via a cold LN_2_ target (∼77 K), the instrument was mounted to fixed plate that elevated it to the level of the BCUs and could be heated via controlled strip heaters and cooled using an LN_2_ reservoir. This plate was designed to simulate the conditions of the spacecraft deck (Fig. 10). A series of shrouds are intermittently filled with LN_2_ via automatically actuated valves to simulate the radiative environment of space. These shrouds were cooled to ∼150K, so as to provide an adequately cold background while minimizing the LN_2_ usage during the eight weeks of thermal vacuum testing. All external monitoring, including instrument power and data, chamber instrumentation and LN_2_ are supplied by vacuum compatible feedthroughs. The instrument was blanketed using the flight MLI thermal blankets to simulate the spacecraft thermal configuration as closely as possible (Fig. 10).

### Software

EMIRS ground support equipment interfaces with numerous devices to aid in the characterization of the instrument and ensure efficient data collection. All data both from the instrument and ancillary hardware were directly recorded into the System Integration and Test database along with a corresponding test id, run number and date. This database could then be dynamically queried to produce processable datasets and trend long-term instrument performance through the system integration and test program.

EMIRS data were processed using an open source software package called davinci (http://davinci.asu.edu) which is developed and maintained by Arizona State University. This software package has a long history of spaceflight calibration and data processing pipeline applications, including instruments such as TES, Mini-TES, THEMIS, and OTES. The development of the System Integration and Test pipeline heavily leverages previous development programs and relies on the System Integration and Test database to query and return the desired data in a format that can be easily ingested and processed in davinci. The database software first decompresses the EMIRS data. Then using run numbers, version numbers and test names identifies unique test sets. These test sets are then packaged in a Hierarchical Data Format 5 (HDF5) file, which is a data format that enables structures of data and can associate a range of metadata. This HDF5 format is used as the primary interchange format for EMIRS test and flight pipelines. Following ingest into davinci, a Discrete Fourier Transform (DFT) is performed on all of the interferograms available, poorly behaved spectra resulting from benchtop or chamber vibrations are identified and removed from the processing chain. Additional software identifies the internal and external/scene targets based on the mirror position telemetry, stage position and interferogram signal levels. The user may override these identifications via custom input file. Once similar targets are identified, they are grouped for further processing and additional external telemetry points are attached to these groups (e.g. calibration target temperatures, collimator azimuth/elevation, chamber state, etc.). The EMIRS interferograms are well behaved and during the System Integration and Test program were not separately treated for the “forward/backward” scan directions, though this capability has been added to the flight processing pipeline (see §7.2 for details).

## EMIRS Development Program

### Flight Instrument Development

The EMIRS development program formally began in January 2015 with the definition of the EMIRS concept. Following a Mission Concept Review (MCR) in February 2015, the EMIRS preliminary design began in under the direction of the United Arab Emirates to enter into Phase B. The EMIRS System Requirement Review and System Definition Review (SRR/SDR) occurred on August 7, 2015. Phase B lasted ∼13 months with the Preliminary Design Review (PDR) being held on April 21-22, 2016. Phase C lasted ∼11 months and culminated in the Critical Design Review (CDR) on March 13-14, 2017. The total development length, including mitigation of development issues discussed in §5.2 was ∼55 months, ∼9 months longer than the initial planned development timeframe. The EMIRS instrument was delivered to the LASP on September 27, 2019 and installed on the S/C on October 6, 2019. In order to minimize development duration, the EMIRS program was structured after a protoflight development program with qualification testing of key sub-components and overall instrument qualification to protoflight levels at the end of the development program.

### Development Issues and Outcomes

The EMIRS program encountered several development issues that delayed the delivery of the instrument to the S/C. Because of these delays the EMIRS instrument Engineering Model (EM), which included roughly 90% flight components was delivered to LASP on November 26, 2018 and installed on the S/C. This flight-like instrument enabled EMIRS to undergo certain spacecraft-level tests, including functional testing, shock, S/C-level EMI/EMC and the mass properties tests. For acoustic and sine vibe testing, the EMIRS mass model was substituted onto the spacecraft. The EMIRS flight instrument was installed for the S/C TVAC and pointing alignment testing.

The first significant delay of the EMIRS development program was due to an infrared DLaTGS detector thermal over-test. At an outside test facility, the entire set of EMIRS flight detectors were accidentally subjected to 125 °C. This violated the AFTs for these detectors (80°C). New detectors were procured over the next several months and the EMIRS engineering model detectors, which came from the same lot as the flight detectors and had the same quality, became eligible for flight use. This resulted in a three-month schedule delay and a temporary degradation of the EM fidelity due to limited detector supply.

While recovering from this anomaly, a GIDEP alert was issued in August 2018 on the EMIRS Xilinx Vertex V FPGA, which occurred after the completion of the EMIRS electronics control boards. This GIDEP required the removal, replacement, and rework of the FPGA on the finished EMIRS control boards, again resulting in an additional four-month schedule delay. This GIDEP resulted in the temporary loss of the EMIRS flight spare control board while the various FPGA and nearby components were replaced that were damaged in the removal process. After recovering from the FPGA anomaly and following the integration of the electronics with the EMIRS optical and mechanical hardware, the performance of the off-axis detectors of EMIRS was lower than anticipated. Investigations into the off-axis performance of these detectors to date have yielded inconclusive root causes. Given the EMIRS instrument can meet the Level 1 Science requirements of the EMM mission with a single-on axis detector; therefore, due to the already pressing instrument schedule, a project-level decision was made to forego any additional investigations and rely on the single EMIRS detector that meets performance over the full spectral range. While other detectors do meet performance specifications over subsets of the spectral range (<∼15 μm) and may not be useful for the suite of atmospheric investigations, they can be used to derive reliable surface temperatures to within the specified uncertainties (Table 3).

During the first EMIRS TVAC test, a degradation in the IRF larger than expected was observed during temperature transitions. The root cause of this was narrowed to three potential causes: 1) The interferometer plate flexing more than anticipated, 2) the vibration isolation grommet spacing and stack up, and 3) the fixed mirror mount design. Each of these issues cause a mis-alignment of the fixed-mirror, moving-mirror, and beamsplitter, resulting in non-parallel ray paths through the two arms of the interferometer. This interferometer mis-alignment results in a loss of modulation efficiency that has a strong wavelength dependence, as is observed in EMIRS data (Chamberlain 1979). An optical model of the instrument that incorporates mirror tilt was developed to predict the instrument response as a function of misalignment and accurately reproduces the measured instrument response function and mirror tilt to within ±1 arcsecond.

After conducting gravity flip tests, where the interferometer plate was held normal to gravity in both positive and negative orientations, measurements directly indicated both excess interferometer plate flexing and an incorrect grommet stack up. The repair of the grommet stack up, which was stiffened to eliminate motion while continuing to provide vibration isolation, removed ∼10 arcseconds of interferometer tilt. The EMIRS interferometer plate redesign, where a double-sided skin with an isogrid interior was fabricated, removed an additional ∼10 arcseconds of tilt in the system and dramatically improved the performance over temperature. The final source of interferometer tilt is likely temperature-dependent shift of the fixed mirror, which may include up to ∼10-15 arcseconds of tilt over the 0-35 °C EMIRS allowable flight temperature range, with the largest values of tilt and thus performance degradation occurring at the lowest temperatures (and shortest wavelengths). A corrective action to replace the fixed-mirror mount with an entirely new design was schedule prohibitive from a S/C integration perspective, a program level decision was made to add two 5.6 W operational heaters that will raise the instrument temperature by ∼7-10°, moving it away from the high-tilt (>10 arcseconds) regime and allow EMIRS to meet its performance requirements over its full wavelength range. This rework and root cause investigation that included TVAC activities resulted in an additional two month schedule delay. The instrument then underwent a final TVAC test to complete the radiometric calibration (§5.3.4) and complete the required number of thermal cycles.

### Environmental Test Summary

#### EMI/EMC Testing

EMIRS was subjected to a set of Electromagnetic Interference and Compatibility tests. The instrument underwent conducted emissions (power leads), in-rush testing, conducted susceptibility (power leads) and radiated emissions testing following the General Design Requirements of the mission. For all of the tests where the susceptibility of EMIRS to interference was being tested, the instrument housekeeping was actively monitored for any packet errors or anomalies, and none were found. For these tests the instrument was placed in a largely quiescent mode. For the radiated emissions testing, EMIRS was placed in to a “noisy” mode where all motors were routinely actuating, science was being collected and the largest power draws were present. EMIRS radiated emissions were ∼50x below the requirement for the mission.

#### Vibration Testing

The EMIRS instrument underwent vibration testing to proto-flight levels for the Mitsubishi Heavy Industries H2A Launch vehicle. This testing was carried out at an outside facility (Grey’s Engineering, Tempe, AZ) and included both random and sine vibration test profiles. In the direction nominal to the mounting surface, the EMIRS instrument underwent a 10.3 Grms random vibration and in the lateral directions a 7.6 Grms load. Sine vibration loads were ramped ranged from 5 Hz at 0.154 G to 50 Hz at 12.5 G. In order to ensure a successful test, three risk reduction tests were carried out to sub-system levels. First the EMIRS mass model was tested to ensure a good correlation between the Finite Element structural Model (FEM) of the interface plate and the EMIRS instrument. Then the EMIRS pointing mirror assembly (excluding the actuator) were taken to qualification levels to characterize the modes of the baffle and Peek snubber material performance. Finally the Interferometer Assembly underwent a risk-reduction test to characterize the grommet isolation behavior and the sensitivity of the moving mirror assembly. Following the rework of the interferometer plate, the instrument was subjected to two more vibration tests, one at the same proto-flight qualification prior to thermal vacuum testing and one additional vibration test at lower levels (more in line with actual launch loads without margin) to ensure the instrument would continue to perform well after launch.

#### Collimator Field of View Testing

To determine the instrument Field of View (FOV), Scan Field of View (SFOV) and Instantaneous Fields of View (IFOV), EMIRS was mounted to a rotary stage in front of a 8″ aperture collimator on azimuthal and elevation articulation stages, with an automated shutter. As with the TVAC testing, all data acquisition was fully automated with commandable step sizes synchronized with EMIRS data acquisition. The LabView interface uses a software loop to independently position each axis to the user specified angle. The software loop makes a coarse move based on the collimator step position, reads the position from the absolute sensor, then refines the position by repeating the move + read absolute position. The loop terminates when the axis is within a margin of error. These data were time tagged and stored in the System Integration and Test database where the data could be retrieved together for easy downstream processing. Additional collimator testing was carried out where the full aperture of the instrument was illuminated with a source and a single arm of the interferometer blocked and was fed with a chopped signal optimized for the frequency response of the detectors. This permitted an assessment of the modulation efficiency of the instrument, a key metric for evaluating the performance of the interferometer.

#### Thermal Vacuum Testing

**Fig. 11:**
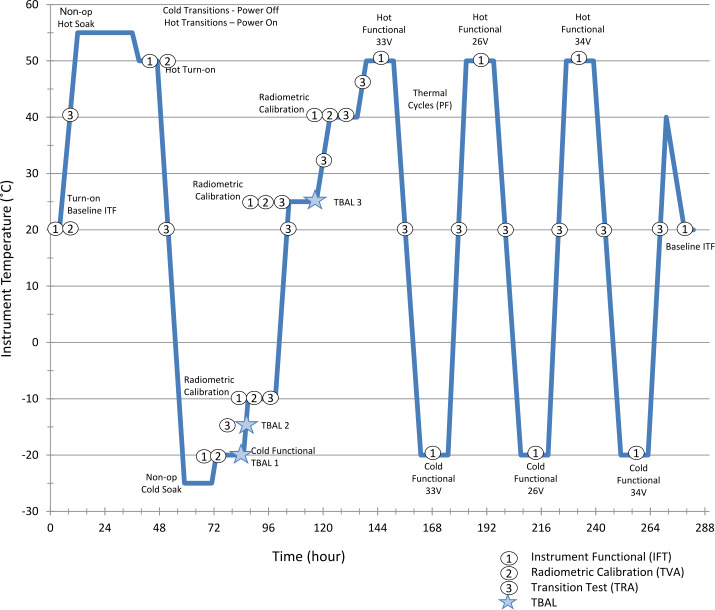
Nominal EMIRS Thermal Vacuum test profile

**Fig. 12:**
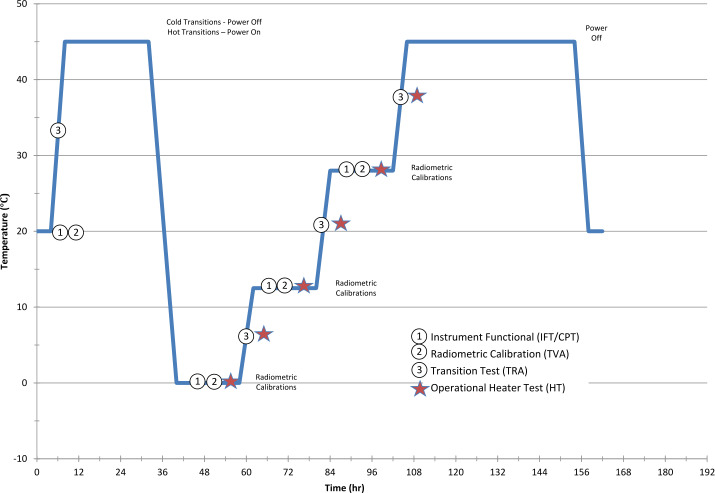
EMIRS Thermal Vacuum test profile for operational heater and interferometer plate rework verification

EMIRS was radiometrically calibrated over a range of instrument temperatures. Specifically, the initial test profile (Fig. 11) was set to provide numerous instrument thermal cycles to verify workmanship and 5 calibration plateaus, -20 °C, -10 °C, +25 °C, and +40 °C with 5 scene temperatures, 150 K, 220 K, 300 K, 340 K, and 380 K with one of the targets being set to 85 K to simulate space observations. This range of scene temperatures encompasses the beyond the expected martian surface temperature range and was carried out at every calibration plateau. In an additional TVAC test following rework of the interferometer plate to correct alignment issues over temperature and the addition of operational heaters to bias the instrument to warmer temperatures to further improve the instrument response function (Fig. 12). When this thermal profile was completed, the instrument had been tested at 0 °C, +12.5 °C, and +27.5 °C, adding 3 additional full calibration plateaus to the original 5, resulting in 40 instrument calibration activities during thermal vacuum testing. During all transitions to warmer temperatures, the instrument was powered on and continually collected data. These transitions typically lasted 3-5 hours providing additional instrument over temperature performance data.

### Post-Delivery Integration

Following the delivery of the EMIRS instrument to the University of Colorado Boulder’s Laboratory for Atmospheric Space Physics for integration onto the spacecraft. The instrument was integrated, a CPT and IFT were run to ensure all electrical interfaces were performing as expected. Spacecraft thermal vacuum testing began ∼2 weeks later, where the instrument and spacecraft underwent additional thermal cycles. The instrument was powered for the majority of the testing with LPTs occurring before and after the test program and multiple times throughout the TVAC program. Following the TVAC testing, the S/C was shipped to Dubai to undergo long-duration testing. In this scenario, weeks long activities tested the science planning products in simulated science orbits. CPTs and IFTs along with detailed visual inspections were performed after every major ship event. During this long duration testing, the COVID-19 pandemic became a global reality, causing significant schedule and personnel pressure throughout the program (Amiri et al. 2020, this volume). Instrument staff were originally planned to be on site thorough the launch campaign; however, due to quarantine requirements and difficulties traveling, a skeleton crew traveled with the S/C from Dubai, UAE to Tanegashima, Japan and nearly the same crew remained through launch, augmented by additional hands-on required specialties. Remote operations and inspections validated the final instrument performance prior to close out and launch (Amiri et al. 2020, this volume).

## Pre-Launch Instrument Performance

### Gain

**Fig. 13:**
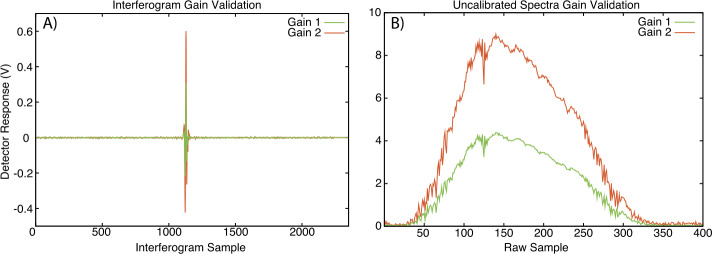
EMIRS gain example from a 4-second, 5 cm^−1^ CPT with a 60 °C blackbody target under ambient conditions

The EMIRS instrument has two gain settings. These gain settings are selectable in the data acquisition command and though the gain 2x state is tuned to ensure that the entire dynamic range of space, calibration and Mars are observable without saturation or undersaturation. The gain was determined in thermal vacuum testing for multiple instrument temperatures. The largest signal EMIRS will observe is that of space when the instrument is warm, while data of the internal calibration target and Mars produce smaller signals, as the detector is measuring the radiance difference from itself. Therefore, when a target temperature is close to the detector temperature, a smaller signal is generated. For each instrument temperature, the peak-to-peak (corresponding to the ZPD) interferogram voltage signal was measured in both gain 2x and gain 1x. Approximately 100 samples (excluding interferograms with thermal vacuum chamber noise present) at each gain state were acquired and averaged together with a stable instrument and target. The ratio of these values is the instrument gain (Fig. 13). The EMIRS gain is determined to be 1.9938 ± 0.0058 and is therefore assumed to be precisely 2 as designed.

### Field of View

The EMIRS field of view was measured in azimuth and elevation using the 8” aperture collimator while observing a glowbar target. The EMIRS alignment cube was referenced to the boresight of the instrument using a periscope viewed through the collimator and an internal instrument reference.

**Fig. 14:**
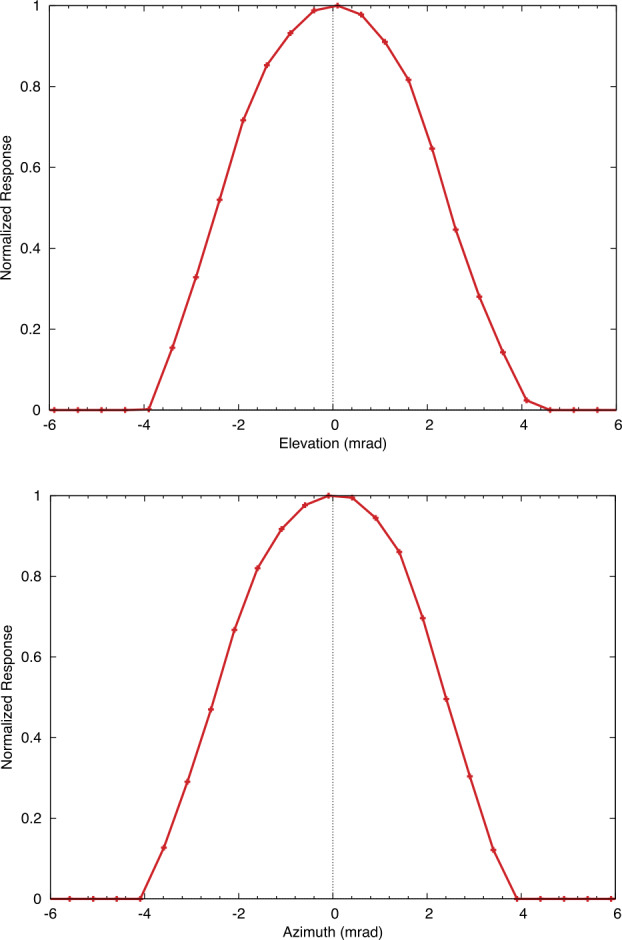
EMIRS center detector instantaneous field of view for the elevation (top) and azimuth (bottom) for the on-axis detector

In order to determine the field of view, the target was viewed through a 1-mrad wide vertical or horizonal slit placed at the collimator focus. During testing, the collimator was oriented on the EMIRS optical axis in each direction, then the collimator was moved in small steps (∼0.5 mrad) in either azimuth or elevation for 2.5 mrad beyond the expected edge of the FOV. This was completed for each detector and the full field of view of the instrument. At each location, 20 interferograms were collected and a closed shutter set of 20 interferograms were also collected to remove any thermal drift in the system due to viewing the warm glowbar target. These 20 interferograms were averaged at each location and corrected for the thermal drift determined based on the closed shutter data. The peak to peak (minimum and maximum) interferogram values for all observations were normalized to 1 and compared against collimator position. This process was repeated throughout the system integration and test program after each major environmental test. The data shown in Fig. 14 were measured following environmental and thermal vacuum testing, immediately prior to the delivery of the instrument to the spacecraft.

### Encircled Energy

**Fig. 15:**
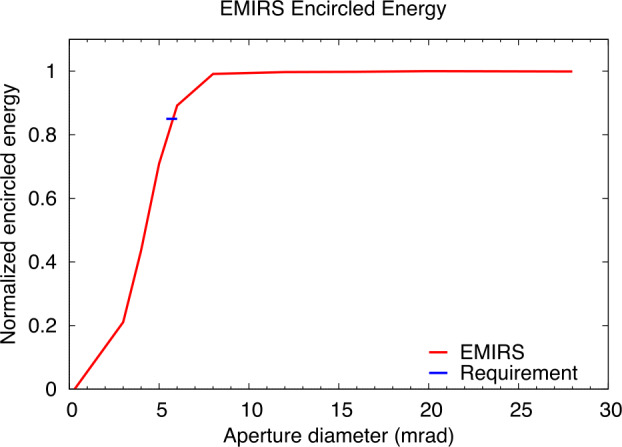
EMIRS encircled energy performance that defines the field of view for the on-axis detector. The blue line indicated the EMIRS requirement of >85% of the energy within the 5.5±0.3 mrad instantaneous field of view

The EMIRS encircled energy, which is a measure of the light captured by the detector at a given aperture that defines the point spread function of the optical system, was also measured and defines the field of view of the instrument. For each aperture diameter, 30 spectra of the collimator glowbar target were collected followed by 30 spectra of the shutter, again to remove thermal drift from observing the warm glowbar target. The peak to peak interferogram values were averaged to maximize the signal to noise at each location (Fig. 15). Based on these data and the encircled energy requirement of 85%, the EMIRS geometric footprint is determined to be 5.5 mrad.

### Spectral Sampling and Spectral Range

The EMIRS spectral sampling is defined by the total travel distance of the Michelson interferometer moving mirror. As described in §3.3.2 the laser metrology wavelength determines the total travel distance and the number of samples collected in each interferogram. The nominal wavelength of the EMIRS laser at 25 °C is 0.846 μm. In order to determine the exact wavelength of the laser, the spectra of the laboratory hot-plate calibration target were observed at long path lengths where the atmospheric constituents (CO_2_ and water vapor) which have sharp spectral features were measured. Following lessons learned from the OTES development program, it was found that best-fit spectral matches to these atmospheric constituents were as reliable, and more straightforward to measure than polyethylene. These atmospheric transmission spectra were compared to MODTRAN modeled spectra (Berk et al. 2014) for the given laboratory conditions and the wavelengths of the water vapor and CO_2_ spectral bands matched the measured EMIRS spectrum within the measurement error, confirming the interferometer spectral sampling. The laser wavelength is known to shift with temperature (∼0.06 nm/K). Over the EMIRS operational temperature range extremes this translates to a shift of ∼2 nm, translating to a spectral sampling difference of ∼0.02 cm^−1^, which is negligible for the EMIRS 5 cm^−1^ and 10 cm^−1^ modes.

### Internal Calibration Target Properties

In order to determine the properties of the EMIRS internal calibration target, a two temperature, full aperture calibration was developed for the two external BCU targets during thermal vacuum testing. This calibration was then used to derive the spectral radiance of observations of the internal calibration target. Then a series of Planck radiances were computed using the temperatures of the 4 calibration target thermistors and assumed emissivity of 1. Emissivity and the average temperature of the calibration target thermistors were then varied in increments of 0.001 and 0.1 K respectively with new Planck radiances calculated for each combination. The best fit of the measured calibrated radiance to the modeled Planck radiances is that of a 0.3 K temperature offset and a 0.002 emissivity offset. This analysis was carried out with an instrument temperature of 14.3 °C (within the 10-40 °C instrument performance in specification AFT) and BCU target temperatures of 300 K and 77 K. This knowledge of the calibration target properties is incorporated into the instrument calibration equations (Eq. (5)).

### Instrument Response Function

**Fig. 16:**
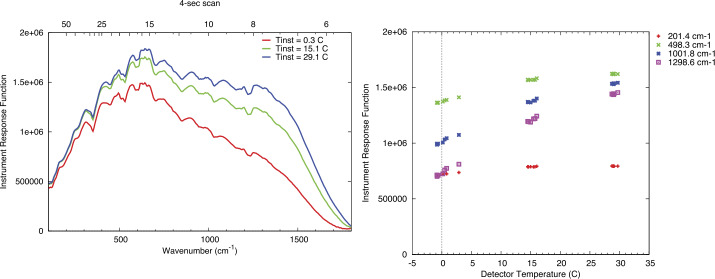
Representative EMIRS Instrument Response Function for 3 different temperatures (left) in W Volt cm^−2^/cm^−1^ sr^−1^. The variation in Instrument Response Function (right) over measured detector temperature at 4 representative wavelengths. These data are a representative set of data collected in the last instrument thermal vacuum test program

The EMIRS instrument response function (IRF) describes the instrument transformation from measured raw-voltage interferogram as measured by the detector. This conversion accounts for all of the optical components (e.g. mirrors, coatings/optical finishes, beamsplitter, lenses, detector performance, etc.) and their variations with wavelength. The IRF varies in amplitude and shape over instrument operational temperatures (10-40 °C) due to several factors including the detector performance which is reduced at lower temperatures and second the alignment of the interferometer, specifically the fixed mirror. The representative IRFs (Fig. 16) were acquired during instrument thermal vacuum testing (Fig. 12). These data were computed using one of the TVAC BCU calibration blackbody standards and the internal EMIRS calibration target. The EMIRS instrument IRF for both 2 and 4 seconds are independent of sampling mode and thus the IRFs are nearly identical. The EMIRS IRF will be computed for each observation of Mars using observations of space and the internal calibration target acquired before, during, and after each observing sequence. In addition to their use in calibrating the Mars data, these IRFs will be monitored throughout the mission to verify that the instrument performance does not change over time.

### Precision: Noise Equivalent Spectral Radiance

**Fig. 17:**
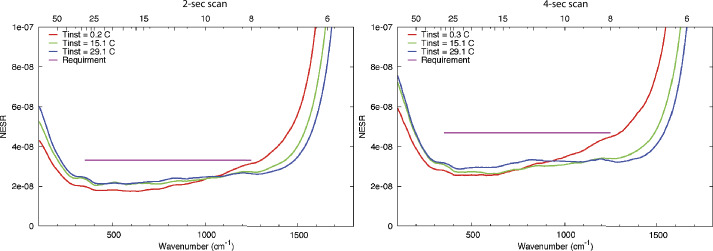
EMIRS Noise Equivalent Spectral Radiance (NESR) precision plots. These figures illustrate the variation in instrument performance over temperature for both 2 (left) and 4 (right) second modes respectively. The lowest instrument temperatures (0 °C) provide the lowest performance likely due to a mechanical tilt movement of the EMIRS interferometer fixed mirror of <10 arcseconds. Due to the operational heaters mounted on the interface plate, the EMIRS is predicted to never dip below ∼10-15 °C and will always remain within ∼10-45 °C

**Fig. 18:**
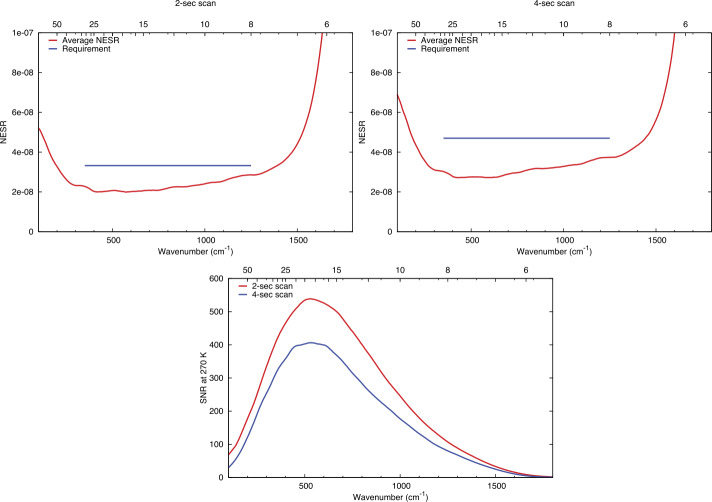
EMIRS average NESR (top) for both 2 (left) and 4 (right) second modes respectively. The corresponding Signal to Noise Ratio (bottom) is shown for a scene temperature of 270K for both 2 and 4 second modes. Instrument performance as compared to scene temperature is provided in Fig. 19. Note: 4-second/5 cm^−1^ mode provides lower SNR as spectral sampling 2x finer while the integration time 2x longer, resulting in a net sqrt(2) difference

The measure of the instrument noise and measurement precision is defined by the instrument NESR. The instrument NESR is a key metric trended throughout the development process as it provides an end to end characterization of instrument performance. However, measuring the NESR is challenging because a small (<0.1 °C) drift in the instrument or target temperature can cause a 50% increase in the NESR. The NESR in Fig. 17 shows the instrument NESR over temperature as acquired in thermal vacuum chamber activities for both 2 and 4 second modes. The NESR for the 2 second mode is sqrt(2) lower than that of the 4-second mode due to the nature of the integration (random uncorrelated noise). As with the flight calibration pipeline, the NESR is processed in both forward and backward mode independently. While the noise level is equivalent for both forward and backward data individually, the small differences between the forward and backward directions can result in slightly different NESR, therefore we show data from only a single scan direction. This was also observed in OTES data (Christensen et al. 2018). As the instrument operates nominally from 10-40 °C the requirement is met with significant margin over the full wavelength range required. The corresponding average NESR and SNR are shown in Fig. 18. As with the IRF, NESR will continue to be trended throughout the mission and the first measurements in flight reveal EMIRS is performing as expected and as measured on the ground during system integration.

### Absolute Accuracy

**Fig. 19:**
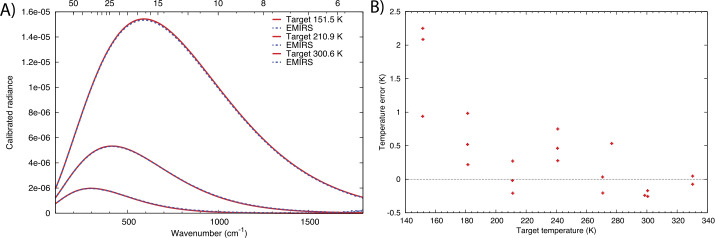
(**A**) EMIRS calibrated radiance in units of W cm^−2^/cm^−1^ sr^−1^ viewing the BCU set to multiple target temperatures, along with the Planck blackbody radiance determined using multiple BCU thermistors. These data were acquired with an instrument temperature of ∼25 C. (**B**) The temperature error as measured by the EMIRS instrument as compared to the target temperature as determined using multiple BCU thermistors

The EMIRS absolute accuracy requirement is <1.5% integrated radiance of an ideal blackbody at a temperature of 270 K. In order to verify this, the calibrated instrument radiance is compared to an ideal Planck blackbody at the temperature measured by the precision BCU target temperature. The EMIRS-measured radiance is an excellent fit to Planck radiance when observing the BCU targets (Fig. 19a). The percent absolute radiance error for a 270 K target is <0.5% over the range of EMIRS operating temperatures, and the EMIRS absolute calibration far exceeds the requirement. The absolute calibration error can also be expressed in terms of the error in the kinetic temperature that is derived from fitting a Planck function to the measured calibrated radiance (Fig. 19b).The maximum error, occurs at the lowest target temperatures (150 K) and is ∼2 K while nominal target temperatures (e.g. >200 K) have errors <0.75 K.

### Linearity

**Fig. 20:**
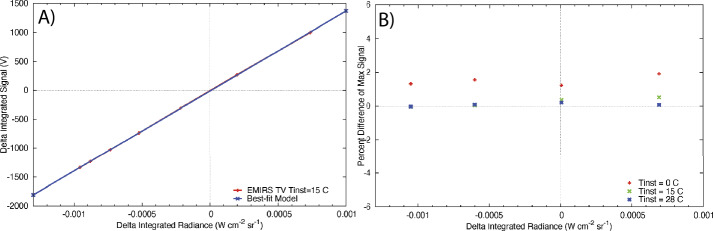
(**A**) EMIRS instrument integrated signal, indicating a linear response between measured signal in voltage and radiance. (**B**) Difference between the instrument measured signal and the best-fit linear model vs delta radiance for 3 different instrument temperatures

The EMIRS linearity requirement is 10% of the full scale. In order to verify this requirement, radiometric calibration data during thermal vacuum testing were acquired over multiple instrument temperatures (0, 15, 28 °C), covering the expected instrument operational range. The measured voltage spectrum was acquired when viewing both the internal calibration target and the external BCU target. These data were integrated from 150 cm^−1^ to 1480 cm^−1^. In order to produce the delta signal the difference between the integrated voltage spectrum of the internal calibration target and the BCU were computed, for the given temperature plateaus. To produce the delta radiance, the difference between the integrated Planck radiances observing the same internal calibration target and the BCU at the same plateaus were differenced (Fig. 20). The result is a linear relationship between delta signal as measured by the instrument to the delta radiance as modeled for the calibration targets. This was completed for multiple instrument temperatures and the maximum deviation from a linear function is <2%.

**Fig. 21:**
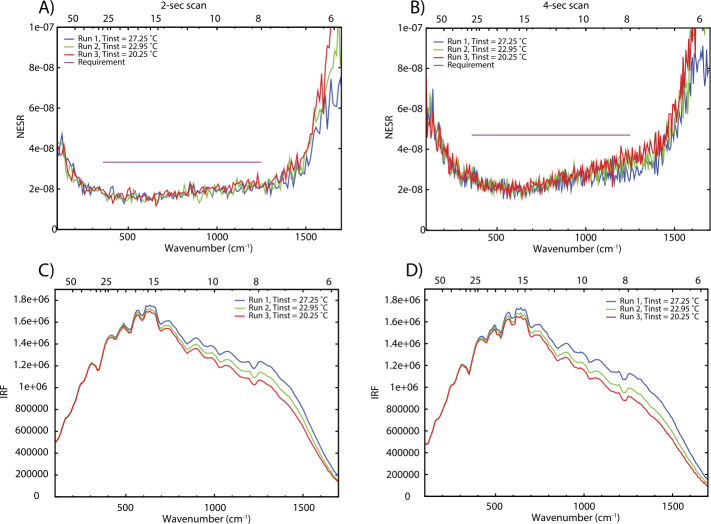
EMIRS cruise performance data indicate the instrument is performing as expected, consistent with ground based calibration. (**A**) 10 cm^−1^. (**B**) 5 cm^−1^ NESR both exceed performance and are nearly identical to the performance data from ground calibration during TVAC (Fig. 17) over the instrument temperatures experienced during cruise (Run1 on 2020-09-27 T13:48:00 (UTC), Run 2 on 2020-11-18 T01:05:00 (UTC), Run 3 on 2021-01-06 T08:50:00 (UTC). (**C**) 10 cm^−1^ and (**D**) 5 cm^−1^ IRFs continue to exhibit the same trends with temperature observed during TVAC testing (Fig. 16)

### EMIRS Performance Trending During Cruise

During the ∼7-month cruise from Earth to Mars, EMIRS carried out a series of routine observations to assess the instrument health and trend instrument performance over time. These activities were carried out 3 times prior to Mars orbit insertion and indicate the EMIRS instrument is behaving as expected from TVAC and ground testing. EMIRS is continuing to exceed all performance criteria which are most readily observed through the instrument NESR.

## EMIRS Concept of Operations

### In-Flight Operations and Observing Strategy

**Fig. 22:**
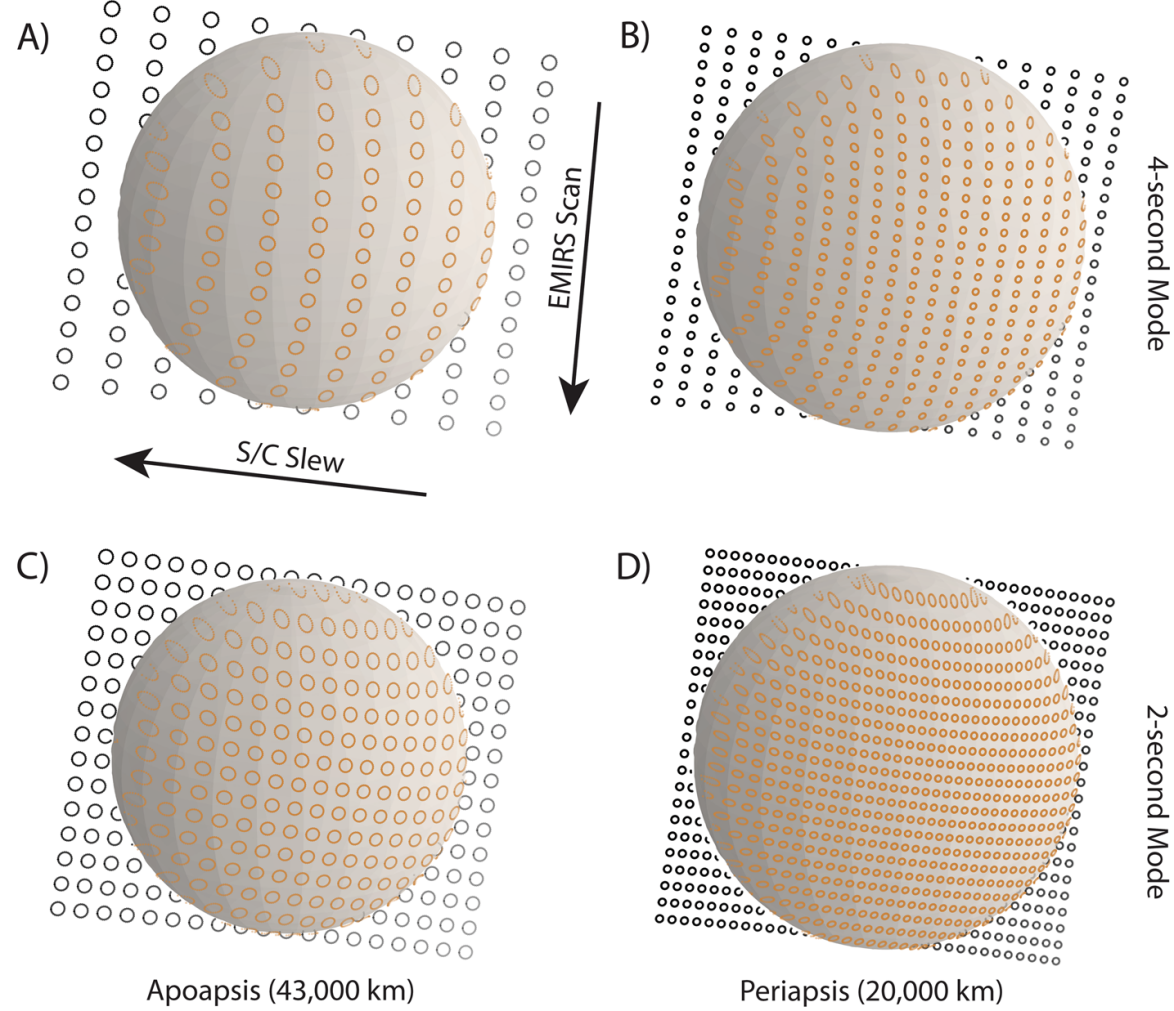
EMIRS R-OS1 (top, **A** & **B**) and R-OS2 (bottom, **D** & **E**) synoptic observation strategies observed. The sphere represents Mars, and the individual circles represent EMIRS center detector observations. These observations are simulated for a S/C altitude of 43,000 km (left, **A** & **C**) and 20,000 km (right, **B** & **D**). Mars is scaled to the same apparent size

EMIRS has two observation strategies (R-OS1 & R-OS2, as shown in Fig. 22) and these observations cover the same total field of view for a given spacecraft altitude although the angular size of the martian disk changes depending on the S/C altitude (Table 6). EMIRS observes half of Mars with resolution less than 300 km and it will acquire ∼60 observations per week (∼20/orbit) depending on the geometry of the solar keep out zone, where EMIRS cannot look back within 13.5° of the Sun when observing the nightside of Mars (Amiri et al. 2020, this volume). After completing a standard internal calibration set of 20 collects, EMIRS will rotate its mirror to the starting position, with a combined 90 seconds for the mirror move and interferometer control servo convergence time. The spacecraft will then initiate a single axis slew across the martian disk, maintaining a constant slew rate according to either the smear limit requirement (∼1/10th of a pixel) or the time it takes EMIRS to complete the full X°×Y° raster acquisition. As the spacecraft slews in one direction, the EMIRS instrument will move its pointing mirror in the orthogonal direction to scan across the planet with a single directional scan (from the top down) and retrace back to the same starting step. On the disk, scans will include ∼2-7 space looks on both sides of the disk. However, in the center of the disk, the number of space looks is reduced in to order to focus on collecting data of the disk. Upon completion of the EMIRS raster, a command is issued to EMIRS to return the pointing mirror to home/internal calibration position and 20 collects of the calibration target are again acquired.

**Table 6:** Summary of EMIRS observation parameters

Observing Strategy	Description
S/C Slew Across Disk:	10.7° – 19.3° based on altitude (apoapsis to periapsis)
Instrument Scan:	8.7° – 16.2° based on altitude (apoapsis to periapsis)
Effective Scan Rate:	Individual spectral acquisitions take 2 and 4 secs for R-OS2 and R-OS1
Slew Rate:	≤0.672 °/min (0.0112 °/s) at periapsis to ≤1.008 °/min (0.0168 °/s) at apoapsis (scan driven)

The total observation takes ∼29 min at periapsis (20,000 km) and ∼11 min at apoapsis (43,000 km). Due to the relatively long orbital period (∼55 hours), the altitude changes at most by ∼50 km over the EMIRS periapsis observation. All EMIRS footprints are mapped onto the disk using the time at which each spectrum is acquired, thus accounting for the small spacecraft altitude variations over the observation duration. EMIRS can also pause its acquisition sequence at the start of each scan “column” in order to support a variety of slew rates. This process enables EMIRS to collect data with minimal gaps over the martian disk.

### In-Flight Calibration Strategy

The EMIRS instrument data will be calibrated in flight using Eq. (5), where observations of space and the internal calibration target bracket observations of the martian disk. Nominally each EMIRS raster is bracketed by a set of 20 interferograms of the internal v-groove calibration target. The temperature of the calibration target is measured in 4 different locations and the average is taken as the temperature of the calibration target. Typically, the deviations of the temperature sensors embedded in the calibration target are <0.1 K. EMIRS also observes space on each side of the disk and the time between space observations is typically <40-80 seconds, while the maximum time between internal calibration observations is ∼11 minutes. Experience from OTES has shown that these intervals (∼10-15 minutes) are sufficient to meet the EMIRS absolute calibration requirement and due to the more frequent periodic space looks (every ∼2 minutes), instrument temperature drift can be removed during calibration. All interferograms are uncompressed and processed through a Discrete Fourier Transform (DFT). Following this transformation, space, target and internal calibration observations are identified both by the reconstructed geometry, but also the mirror position and signal levels. The calibration observations (space and internal calibration) are then individually grouped and any trends between the internal calibration groups and individual space observations will be linearly interpolated to provide the most reliable trending of instrument temperature changes. While the instrument temperature will vary slightly over the course of an observation set, thermal modelling and testing indicate that the temperature will vary nearly linearly at <0.1 °C/minute, resulting in <∼1 °C variation over the time between internal calibration observations and ∼0.1 °C between space observations.

If a set of calibration targets are not available, the closest calibration target observation is used. Alternatively, pre-flight IRF at the appropriate instrument temperature can be used to derive the scene radiance if necessary. This approach, where only a single point (e.g. space) and a pre-flight IRF would be used in an anomaly or fault mode for the instrument. Observations calibrated in this manner are not likely to be as accurate as the nominal 2-point calibration, and if necessary, will be determined later in flight. This approach is similar to the heritage approach of OTES (Christensen et al. 2018). The experience with previous instruments such as OTES, TES and Mini-TES indicates that while the instrument response varies over temperature (Fig. 16), it is highly repeatable and stable over the life of the life of the instrument (Christensen et al. 2001, 2004). Therefore, if for some reason an anomaly prohibits the acquisition of an internal calibration target, evidence from previous programs indicates radiometric calibration of data will still be possible.

## Data Processing and Archiving

### Summary of EMIRS Processing Pipeline (Level 0-Level 2 and Level 2-Level 3)

**Fig. 23:**
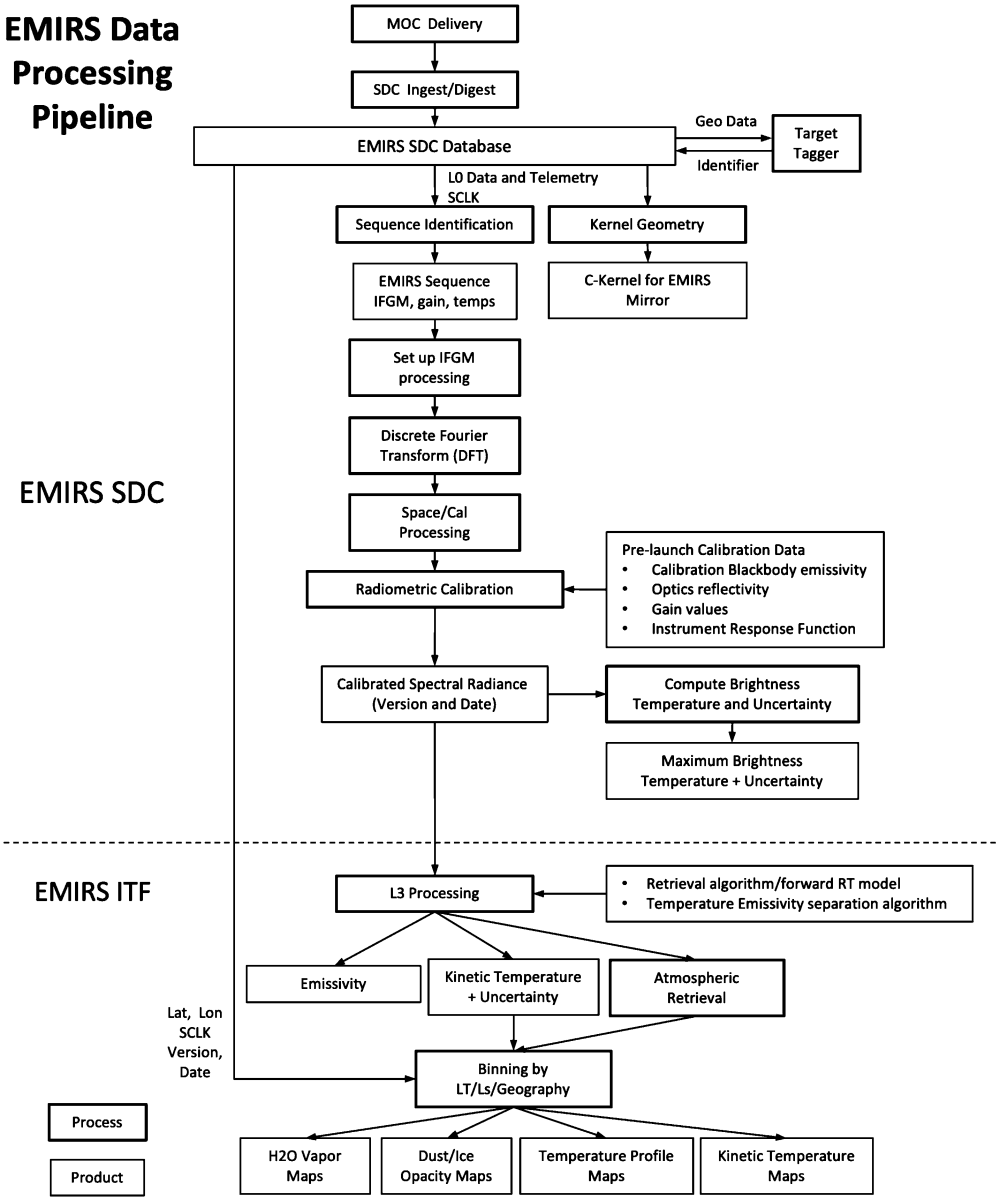
EMIRS L0-L3 processing flow chart that illustrates processes/algorithms and products. The L0-L2 instrument pipeline (including processing to calibrated radiance) occurs in the EMM Science Data Center automatically upon receipt of data. The L2-L3 process occurs at the EMIRS ITF institutions (ASU/NAU) and final products are delivered to the EMM SDC after validation

The EMIRS processing pipeline (Fig. 23) is split between the Emirates Mars Mission Science Data Center (SDC) and the Instrument Team Facilities (ITF) (Amiri et al. 2020, this volume). L0 data are delivered from the Mission Operation Center (MOC) to the SDC. At this point the automated EMIRS pipeline is notified of new data to process. All telemetry and science data are ingested into the EMIRS PostgreSQL/PostGIS Database where data is then split into observation sequences. Each observation sequence is bracketed by a series of internal calibration observations and space-looks throughout the acquisition. The Target Tagger process identifies each type of EMIRS data and marks the individual spectra. The geometry produced as a part of this process, is converted in to a NAIF/SPICE C-Kernel which is also delivered as a part of the EMIRS data product generation.

Following the identification of observation sequences, the interferogram processing occurs where they are converted to spectra through a Discrete Fourier Transform (DFT). Following this conversion bad or noisy spectra (due to servo noise, etc.) are identified and removed from further processing. These data are then grouped by space/cal/target and an instrument response function and conversation factor is generated as described in §4. Following the radiometric calibration, L2 products are generated and include calibrated radiance, brightness temperatures versus wavelength, a footprint and geometry (e.g. incidence angle, emission angle, local time, etc.) per detector as well as a host of quality control flags. Quick-look products that illustrate the geometry of the acquisition and are produced for several brightness temperatures. These data are deposited in the SDC repository. The first complete processing of L2 data is expected to occur withing hours of downlink and will continue to be refined over the coming weeks. As additional data are downlinked (e.g. science packets, navigation/kernel data, etc.), observation sequences are tagged with the updated data and the complete pipeline run again for those sequences, with the final data being delivered for distribution 2 weeks after the last expected modification (roughly 4 weeks from the initial downlink, given the EMM retransmit strategy).

Following the generation of L2 data, nightly the EMIRS ITF synchronizes the L2 products to the ITF, where they are staged for L3 processing and validation. The L3 pipeline converts calibrated radiance to observable quantities, such as the aerosol opacity, atmospheric temperatures, water vapor abundance, surface kinetic temperature (derived following the same methods as TES where the atmosphere is clear and surface emissivity is maximal, Christensen et al. 2001), and surface emissivity (also derived using the same algorithms as TES, Christensen et al. 2001). This process operates on individual observation sequences, and again stores these newly derived quantities along with quality control constraints, retrieval uncertainties (§8.3), geometry, and observation conditions in the L3 archive products. L3 archive products are stored in the same style as the L2 products where they are grouped by observation sequence and are delivered back to the SDC. All L1-L2 data are stored in the FITS file format (a PDS compliant format) and include a range of metadata associated with the data products.

Following the re-ingestion into the EMIRS processing database, additional L3 products are generated where selected data products (water vapor column abundance, water ice opacity, dust opacity, temperature profiles, surface temperatures) are binned into 8 local time groups over roughly 5° Ls for the entire globe. These quantities are extracted directly from the database rather than the individual observation sequences, so that selective individual spectra can easily be queried against L2 quality control constraints, geometry, local time, etc. without searching through numerous individual observation sequence files. The binned maps are produced in the Simple Cylindrical projection and are delivered back to the SDC following processing at the ITF. L3 data are also stored in FITS file formats, though binned maps are stored in geoFITS format for ease of ingestion into common mapping tools.

### Summary of Retrieval Description (L2-L3)

The algorithm used to retrieve atmospheric temperature, aerosol optical depth, and water vapor column abundance is based on that used for TES (Conrath et al. 2000; Smith 2004), but updated in a number of ways to improve performance and accuracy. In particular, the forward model portion of the code, which computes an expected EMIRS spectrum given a set of atmospheric state input parameters has been updated to include aerosol scattering. A full multiple-scattering treatment is included using a discrete-ordinates formulation (e.g., Goody et al. 1989; Thomas and Stamnes 1999), so that the retrieved aerosol optical depth values are now full extinction values rather than the absorption-only optical depth retrieved using Smith (2004). Gas absorption from CO_2_ is computed using the correlated-k approximation (Lacis and Oinas, 1991) and aerosol particle size distributions and scattering properties are taken from TES and mini-TES analysis (Wolff and Clancy, 2003; Wolff et al., 2006). The new forward model also takes into account the variation of emission angle across the EMIRS footprint, breaking the footprint into smaller sub-pixels when needed to accurately model the radiance observed by EMIRS. The forward model has also been optimized to produce reliable results over the complete diurnal cycle and seasonal coverage that is possible with EMIRS observations. For example, the number of radiation streams included to model scattering in the forward model is varied as a function of solar longitude and local time. For the standard L3 data products the atmospheric parameters being retrieved are assumed to be homogenous so that retrieved values are averages across the EMIRS field of view.

The spectral signatures of CO_2_ (used for retrieval of the temperature profile), dust aerosol, water ice aerosol, and water vapor are all spectrally distinct (Fig. 1). The algorithm takes advantage of this to use a sequential, iterative approach to retrieve different quantities, which is efficient compared to a full simultaneous retrieval of all quantities at once. A constrained linear inversion routine based on TES and Mini-TES experience (Conrath et al. 2000; Smith 2004, 2006; Smith et al. 1996, 2004) is used to find the retrieved properties that provide a minimum chi-squared difference between the computed and observed spectra. Surface temperature and the atmospheric temperature profile as a function of height are retrieved first using the 15- μm CO_2_ absorption band. In two separate additional steps, the dust and water ice aerosol optical depths are fit, followed by water vapor column. The entire three-step retrieval process is then iterated until convergence, which is typically rapid given the relatively limited interaction between different quantities. Quality control metrics (e.g. rms difference between observation and best-fit model radiances) are used to quantitatively evaluate the quality of the final retrieved values. All retrieved quantities and quality metrics are included in the Level 3 data products from EMIRS (Fig. 23).

### Retrieval Verification and Uncertainties

The verification of the forward model performance (key to the retrieval) was carried out first through validation against MGS-TES data. However, TES observations (even when including aerobraking observations) do not encompass the range of geometries, seasons, local times etc., so a hybrid approach was developed that uses the Laboratorie de Meteorologie Dynamic (LMD) Mars Climate Database (MCD) data and MGS-TES aerobraking observations. This comparison was made in order to expand the parameter space to that which EMIRS and EMM will cover given the unique orbit.

**Fig. 24:**
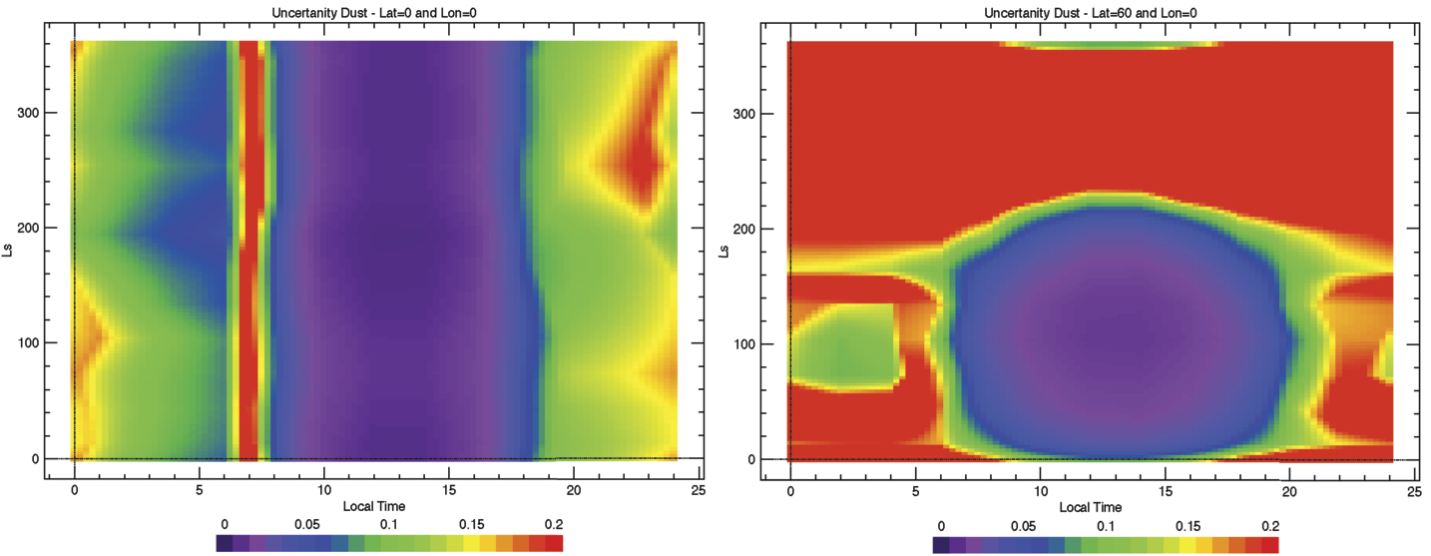
(Left) the expected uncertainty in retrieved dust aerosol optical depth at the equator as a function of season and local time for retrievals at 0° E, 0° N and (right) 0° E, 60° N

As described previously in §3.2.2, the retrieval uncertainty is estimated numerically by computing the rms change in retrieved quantities given the measured noise level of the instrument. This methodology is actively utilized to guide observation plans and to indicate the range of environmental and observational parameters where useful retrievals can be obtained. Uncertainty modeling is completed for each observable parameter (Table 3), under a range of scenarios, with numerous free parameters (e.g. number of sub-pixels, thermal contrast, L_s_ and local time variations, number of radiation streams in the retrieval’s forward model, column abundances, etc.). As an example, Fig. 24 illustrates the uncertainties in dust optical depth associated with variations in local time and season for two different latitudes. The thermal contrast between the surface and atmosphere is found to be the driver for these uncertainty models (assuming the number of streams and sub-pixels are sufficient) where the thermal contrast and uncertainty are inversely proportional. Under conditions where the surface and atmosphere are nearly the same temperature (e.g. close to a difference of 0 °K), the uncertainties become large. Similar trends as those observed in Fig. 24 exist for other atmospheric constituents, namely water ice and water vapor (Badri et al. 2019).

The time of day where the lowest uncertainties for dust retrievals occurs is between 9:00 and 16:00 pm. While in this equatorial scenario the dominant factor controlling thermal contrast and thus uncertainty is the local time, at higher latitudes the seasonal variations have a larger effect on the uncertainty (Fig. 24, right). The elevated high uncertainty at approximately 7:00 am (Fig. 24, left) is associated with sunrise where thermal contrast is extremely low. Of note is that conditions post-midnight until sunrise have lower uncertainties than those just following sunset, perhaps due to the simultaneous cooling of the atmosphere and surface. Through the remainder of the night, the surface continues to cool, while the atmosphere remains relatively constant. Thus, later in the night a larger surface/atmosphere contrast may be present, resulting in lower uncertainties. Overall, in low-latitude regions, the estimated uncertainty is low and atmospheric properties will be readily retrieved across most of the martian day.

**Fig. 25:**
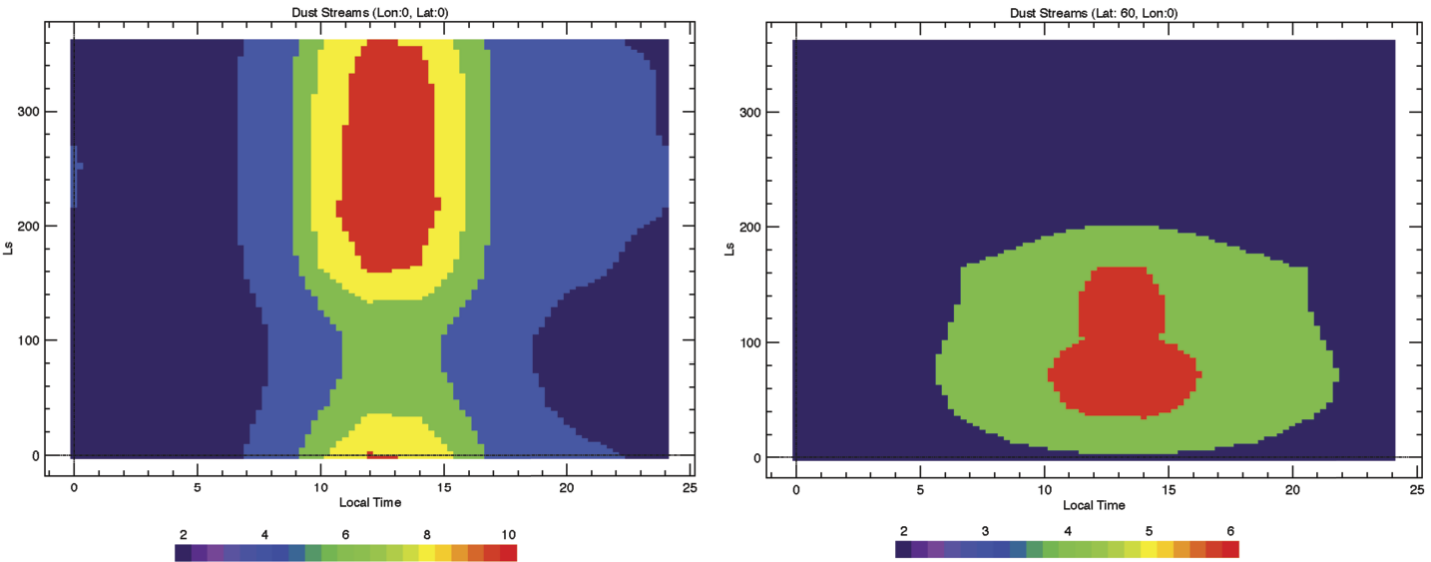
(Left) The variation in the number of radiation streams required to accurately model the observed EMIRS spectrum as a function of local time and season for dust retrievals centered at 0° E, 0° N and (right) 0° E, 60° N

In another scenario, the number of radiation streams was investigated in the discrete ordinates formulation of the forward radiative transfer model to determine how many are needed to accurately model EMIRS observations to within the measured NESR as a function of local time and season. The number of streams needed was obtained by computing the difference in radiance between a reference model using an arbitrarily large number of streams (64 in this case) and models with smaller numbers of streams (2, 4, 6, etc.). This radiance difference was then compared against the instrument noise level to obtain the minimum number of streams that are accurate to within 1/5 of the instrument’s noise level. As seen in Fig. 25, the effect of dust absorption and scattering, and thus the minimum number of streams needed to model that, is greater during the daytime since there is a greater thermal signal as compared to nighttime. Also, scattering is greater in the perihelion season (Ls=180°–360°) as both the incident radiance is higher and the typical dust optical depth is larger (e.g., Smith 2004), and thus more streams are required to accurately model the atmosphere.

### Data Product Description

The EMIRS processing pipeline produces multiple products for archival (Fig. 23). These files are PDS compatible FITS files that contain all necessary geometry and calibration information, along with quality control flags to evaluate the data. Level 2 data products are grouped by observation set (internal calibration, space and target data) are provided as calibrated radiance. These data have geometry polygons that describe the EMIRS footprint as latitude, longitude and radius. Quick-look products of brightness temperatures are also produced at this level. Calibrated spectral radiance at the instrument is the standard data product produced by EMIRS. Additional quantities such as brightness temperatures at each wavenumber, assuming an emissivity of 1 and emissivity are also produced as a part of this calibration pipeline and available in the FITS files.

L3 data products rely on retrieved parameters through the algorithm described in §8.2 to retrieve observable parameters. These products will be re-gridded to 1 pixel per degree (ppd) and map projected into simple cylindrical maps. These maps oversample the highest resolution EMIRS data even at the equator; however they enable the mapping of gradients that would otherwise be lost if the re-binned map resolution were set to the native EMIRS resolution. These data include retrieved parameters (e.g. dust opacity, ice opacity, atmospheric temperature, and water vapor column abundance) as well as derived brightness and surface temperatures. These data are binned according to local time over the previous weeks’ observations (∼60) and use an average combine where pixels overlap, while providing the standard deviation, observation count, minimum and maximum for each pixel in the 1 ppd map.

## Abbreviations

ADC: Analog to digital converter
AFT: Allowable Flight Temperature
ARM: Antireflection microstructure
ASU: Arizona State University
BCU: Bench Checkout Units
CCSDS: Consultative Committee for Space Data Systems
CDR: Critical Design Review
CONOPS: Concept of Operations
CPT: Comprehensive Performance Test
CPU: Central Processing Unit
CVD: Chemical vapor deposited
DFT: Discrete Fourier transform
DLaTGS: Deuterated L-alanine doped Triglycine Sulfate
EMIRS: Emirates Mars InfraRed Spectrometer
EMM: Emirates Mars Mission “Hope”
EM: Engineering Model
FET: Field Effect Transistor
FOV: Field of View
FPGA: Field Programmable Gate Array
FTIR: Fourier Transform Infrared
FWHM: Full-width, half-maximum
G: Acceleration due to gravity (on Earth, ∼9.8 m/s^2^)
GIDEP: Government-Industry Data Exchange Program
GSE: Ground Support Equipment
Grms: Acceleration due to gravity, Root Mean Squared
IFOV: Instantaneous field of view
IFT: Instrument Functional Test
IMU: Inertial measure unit
IRF: Instrument response function
JST: Japan Standard Time
krad: kilorad
kHz: kilohertz
L0: Level 0 Data – raw instrument packets
L1: Level 1 Data – uncalibrated data
L2: Level 2 Data – calibrated data
L3: Level 3 Data – derived physical parameters
LN_2_: Liquid Nitrogen
LVDS: Low Voltage Differential Signaling
MCD: Mars Climate Database
MER: Mars Exploration Rover
MGS: Mars Global Surveyor
Mini-TES: Miniature Thermal Emission Spectrometer
MLI: Multi-Layer Insulation
MO: Mars Observer
NAU: Northern Arizona University
NE$\Delta $T: Noise Equivalent Delta Temperature
NESR: Noise Equivalent Spectral Radiance
OASIS-PS: Operations and Science Instrument Support Planning and Scheduling
OTES: OSIRIS-REx Thermal Emission Spectrometer
PDR: Preliminary Design Review
RMS: Root Mean Square
S/C: Spacecraft
SNR: Signal-to-noise ratio
SDC: Science Data
SRR: System Requirement Review
TES: Thermal Emission Spectrometer
THEMIS: Thermal Emission Imaging System
ZPD: Zero path difference
